# An Antibody from Single Human V_H_-rearranging Mouse Neutralizes All SARS-CoV-2 Variants Through BA.5 by Inhibiting Membrane Fusion

**DOI:** 10.1126/sciimmunol.add5446

**Published:** 2022-08-11

**Authors:** Sai Luo, Jun Zhang, Alex J.B. Kreutzberger, Amanda Eaton, Robert J. Edwards, Changbin Jing, Hai-Qiang Dai, Gregory D. Sempowski, Kenneth Cronin, Robert Parks, Adam Yongxin Ye, Katayoun Mansouri, Maggie Barr, Novalia Pishesha, Aimee Chapdelaine Williams, Lucas Vieira Francisco, Anand Saminathan, Hanqin Peng, Himanshu Batra, Lorenza Bellusci, Surender Khurana, S. Munir Alam, David C. Montefiori, Kevin O. Saunders, Ming Tian, Hidde Ploegh, Tom Kirchhausen, Bing Chen, Barton F. Haynes, Frederick W. Alt

**Affiliations:** ^1^ Howard Hughes Medical Institute, Program in Cellular and Molecular Medicine, Boston Children’s Hospital, Boston, MA 02115, USA.; ^2^ Department of Genetics, Harvard Medical School, Boston, MA 02115, USA.; ^3^ Division of Molecular Medicine, Boston Children’s Hospital, Harvard Medical School, Boston, MA 02115, USA.; ^4^ Department of Pediatrics, Harvard Medical School, Boston, MA 02115, USA.; ^5^ Program in Cellular and Molecular Medicine, Boston Children’s Hospital, Boston, MA 02115, USA.; ^6^ Department of Cell Biology, Harvard Medical School, Boston, MA 02115, USA.; ^7^ Duke Human Vaccine Institute, Duke University School of Medicine, Durham, NC 27710, USA.; ^8^ Department of Medicine, Duke University School of Medicine, Durham, NC 27710, USA.; ^9^ Program in Cellular and Molecular Medicine, Boston Children’s Hospital, Boston, MA 02115, USA.; ^10^ Division of Viral Products, Center for Biologics Evaluation and Research, Food and Drug Administration (FDA), Silver Spring, MD, USA.; ^11^ Department of Surgery, Duke University, Durham, NC 27710, USA.; ^12^ Department of Immunology, Duke University School of Medicine, Durham, NC 27710, USA.

## Abstract

SARS-CoV-2 Omicron sub-variants have generated a world-wide health crisis due to resistance to most approved SARS-CoV-2 neutralizing antibodies and evasion of vaccination-induced antibodies. To manage Omicron sub-variants and prepare for potential new variants, additional means of isolating broad and potent humanized SARS-CoV-2-neutralizing antibodies are desirable. Here, we describe a mouse model in which the primary B cell receptor (BCR) repertoire is generated solely through V(D)J recombination of a human V_H_1-2 heavy chain (HC) and, substantially, a human Vκ1-33 light chain (LC). Thus, primary humanized BCR repertoire diversity in these mice derives from immensely diverse HC and LC antigen-contact complementarity-region-3 (CDR3) sequences generated by non-templated junctional modifications during V(D)J recombination. Immunizing the human V_H_1-2/Vκ1-33-rearranging mouse model with SARS-CoV-2 (Wuhan-Hu-1) spike protein immunogens elicited several V_H_1-2/Vκ1-33-based neutralizing antibodies that bound RBD in a different mode from each other and from those of many prior human patient-derived V_H_1-2-based neutralizing antibodies. Of these, SP1-77 potently and broadly neutralized all SARS-CoV-2 variants through BA.5. Cryo-EM studies revealed that SP1-77 bound RBD away from the receptor-binding-motif via a CDR3-dominated recognition mode. Lattice-light-sheet-microscopy-based studies showed that SP1-77 did not block ACE2-mediated viral attachment or endocytosis, but rather blocked viral-host membrane fusion. The broad and potent SP1-77 neutralization activity and non-traditonal mechanism of action suggest this antibody might have therapeutic potential. Likewise, the SP1-77 binding epitope may further inform on vacccine strategies. Finally, the general class of humanized mouse models we have described may contribute to identifying therapeutic antibodies against future SARS-CoV-2 variants and other pathogens.

## Teaser

A humanized antibody from a recently-developed mouse model potently neutralizes SARS-CoV-2 variants by inhibiting membrane fusion.

## INTRODUCTION

SARS-CoV-2 Omicron and its sub-variants, including the recently emergent BA.5 sub-variant, have spread worldwide and remain the cause of a public health crisis (*1*–*3*). Very recently, yet another Omicron sub-variant, BA.2.75 emerged in India and has begun to spread (*4*–*6*). Omicron-variants have an unprecedented number of mutations in their spike proteins, are resistant to most prior SARS-CoV-2-neutralizing antibodies, and evade antibodies induced by vaccinations (*1*–*3*, *7*–*12*). The few published human monoclonal antibodies that broadly neutralize omicron sub-variants came from B cells of vaccinated individuals and human patients previously infected by SARS-CoV-2 ancestral strains before Omicron (*1*, *13*–*15*). To manage Omicron sub-variants and prepare for potential future SARS-CoV-2 variants of concern (VOCs), new human or humanized antibodies that robustly neutralize all SARS-CoV-2 VOCs are needed. In addition, a characterization of their mechanism of action will be essential.

Binding of the SARS-CoV-2 spike protein to its obligate angiotensin-converting enzyme 2 (ACE2) receptor on the target cell surface initiates infection (*16*). The spike protein is made up of non-covalently linked S1 and S2 subunits (*17*). The receptor-binding-domain (RBD) for ACE2 is located in the S1 subunit, while the S2 subunit anchors the spike protein in the viral membrane. The S2 subunit also contains other sequences that mediate viral/host cell membrane fusion for viral entry. The RBD can adopt two distinct conformations: “down” and “up”; the “down” state is shielded from ACE2 binding, while the “up” state is ACE2-accessible (*18*). Engagement of ACE2 by an “up” RBD exposes the S2’ cleavage site on the S2 subunit to either the serine 2 (TMPRSS2) transmembrane protease at the infected cell surface, or to cathepsin L in the endosomal compartment following endocytosis of the ACE2/SARS-CoV-2 complex (*19*). Cleavage of the S2 subunit by these proteases leads to dissociation of the S1 subunit, which exposes the fusion peptide on the S2 subunit and ultimately leads to fusion pore formation, viral-host membrane fusion, and viral entry into the infected cell (*20*).

Therapeutic SARS-CoV-2 neutralizing antibodies that block virus entry into host cells have demonstrated substantial efficacy for treating COVID-19 infections. The majority of such antibodies target the SARS-CoV-2 RBD and inhibit viral entry by binding to the ACE2 receptor binding motif (RBM), thereby, directly impeding its binding to the ACE2 receptor (*7*). Other such antibodies bind outside of the RBM, but sterically inhibit ACE2 binding (*21*). Some SARS-CoV-2 neturalizing antibodies bind outside of the RBM and do not inhibit ACE2 binding, but can still potently neutralize SARS-CoV-2 VOCs (*7*, *22*–*25*). Several antibodies in this latter class destabilize the SARS-CoV-2 Spike trimer *in vitro,* which has been speculated to be their neutralization mechanism *in vivo* (*24*, *25*). However, the neutralization mechanism of most antibodies in this class is unknown.

A vast portion of the primary antibody repertoire diversity lies in the antigen-contact CDR3-encoding variable region sequences generated *de novo* by non-templated V(D)J junctional modifications during V(D)J recombination (*26*). Indeed, the number of CDR3 sequences that can be generated through junctional diversification exceeds the number of B cells in mammalian immune systems by many orders of magnitude (*27*). Thus, the relative size of the primary BCR repertoire in humans and mice is determined by the number of primary B cells in their immune system (*28*). Immunoglobin (Ig) gene-humanized mice capable of rearranging the full complement of human *Igh* and *IgL* variable region gene segments have yielded many therapeutic monoclonal antibodies, including antibodies that neutralize several SARS-CoV-2 VOCs (*21*, *29*). However, humanized mouse models have not previously been described that yielded monoclonal antibodies that neutralize all of the currently dominant Omicron sub-variants. There could be many possible explanations for the lack of isolation of antibodies that more broadly neutralize SARS-CoV-2 VOCs from Immunoglobin (Ig) gene-humanized mice. One of these possibilities is that mice express only a tiny fraction of the CDR3-diversity present in human primary BCR repertoires, as a mouse has 1000-fold fewer B cells than a human. Early experiments indicated that BCR repertoires generated from rearrangement of a single V_H_ and Vκ could make potent responses to diverse antigens due to immense CDR3 diversification (*30*). Building from this observation, we reported here that a mouse model that generates more human-like CDR3 diversity in association with rearrangement of a single human V_H_ and Vκ could be used to elicit potent SARS-CoV-2 neutralizing antibodies.

## RESULTS

### Single human V_H_- and Vκ-rearranging mouse model for human antibody discovery

**Fig. 1.**
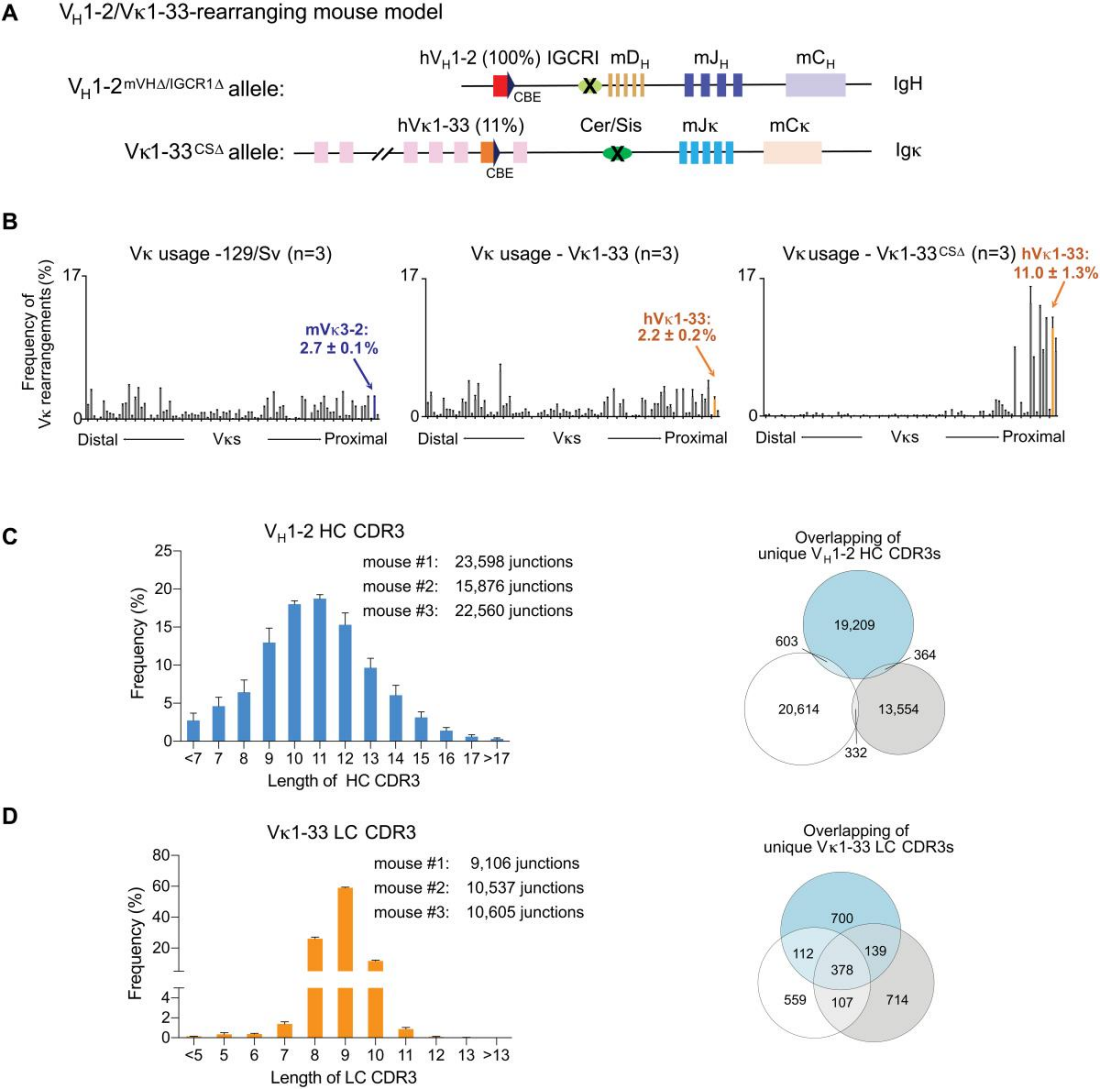
A humanized mouse model with diverse BCR repertoire derived from single human V_H_ and Vκ recombination. (**A**) Schematic representation of modified *Igh* and *Igκ* loci of V_H_1-2/Vκ1-33-rearranging mice. The V_H_1-2^IGCR1∆^ allele was made previously (*33*). We deleted 2 MB region upstream of V_H_1-2 that contained all mouse V_H_s to generate V_H_1-2^mVH∆/IGCR1∆^ allele. We replaced mouse Vκ3-2 with human Vκ1-33 and deleted Cer/sis from the Vκ to Jκ interval to generate Vκ1-33^CS∆^ allele. (**B**) HTGTS-rep-seq analysis of Vκ usage in 129/Sv wild-type (left panel) and Vκ1-33-rearranging mouse splenic B cells in presence (middle panel) or absence (right panel) of Cer/sis. The x axis listed all functional Vκs from distal to the *Jκ*-proximal ends. The histogram displayed percent usage of each Vκ among all productive VκJκ rearrangements. The junction number of each Vκ was shown in Table S1. (**C-D**) Length distribution of V_H_1-2 HC CDR3 (left panel in (**C**)) and Vκ1-33 LC CDR3 (left panel in (**D**)) in splenic B cells. Data were mean ± SD of three libraries from different mice and shown in Table S2. Venn diagram showed the V_H_1-2 HC CDR3 (right panel in (**C**)) and Vκ1-33 LC CDR3 (left panel in (**D**)) complexity. Unique reads derived from the same libraries were in the left panel. Little overlap of V_H_1-2 HC CDR3 sequences among three independent mice indicated tremendous CDR3 complexity.

For these studies, we generated the “V_H_1-2/Vκ1-33-rearranging mouse model” that generates primary BCR repertoires via exclusive rearrangement of a single human V_H_1-2 and predominant rearrangement of human Vκ1-33, with the primary humanized BCR repertoire diversification based on immense CDR3 diversification (Fig. 1A). In this regard, V_H_1-2 is frequently represented in SARS-CoV-2 nentralizing antibodies derived from human patients, but has not contributed to antibodies that broadly neutralize the different VOCs (*31*). For the *Igh* locus in this model, the proximal mouse V_H_5-2 (also known as V_H_81X, (*32*)) was replaced with human V_H_1-2 on an allele in which the IGCR1 regulatory element in the V_H_ to D intervening region was deleted (*33*). Deletion of IGCR1 permits direct cohesin-mediated loop extrusion-based V(D)J recombination scanning to the proximal V_H_ from the J_H_ based V(D)J recombination center (*34*–*36*). Such scanning is impeded at the inserted V_H_1-2 due to its association with the CTCF-binding element (CBE) downstream of the mouse V_H_5-2 (*33*, *34*). Impeded scanning by this linked CBE increases V_H_1-2 accessibility to the V(D)J recombination apparatus and leads to its dominant usage (*34*). We further deleted all functional mouse V_H_s from this allele to avoid any competition with the dominantly rearranged V_H_1-2 (Fig. 1A and Fig. S1A). For the *Igκ* locus in this model, the proximal mouse Vκ3-2, that is expressed in 2.7% of splenic B cells, was replaced with human Vκ1-33, which resulted in its expression in 2.2% of splenic B cells (Fig. 1, A and B; Fig. S1, B and C; Table S1). Then, we deleted the IGCR1-related Cer/Sis element in theVκ-Jκ interval; which, based on prior studies (*37*), we predicted should increase Vκ 1-33 utilization. Indeed, Cer/Sis deletion increased Vκ1-33 utilization to 11% (Fig. 1, A and B; Fig. S1, D and E; Table S1), which we speculate also results from increasing direct V(D)J recombinational scanning to proximal Vκs from a Jκ-based recombination center. We retained the mouse J_H_s and Jκs in this model as their framework sequences outside of CDR3 were largely conserved between mouse and human (Fig. S1F). We also retained the mouse Ds, as mouse Ds are highly related to a subset of human Ds and, in any case, contribution of D segments to CDR3 are often unassignable due to extensive non-templated junctional diversification (*26*).

In the resulting V_H_1-2/Vκ1-33-rearranging mouse model, human V_H_1-2 and Vκ1-33 contributed to the BCRs expressed by 100% and 11%, respectively, of splenic B cells (Fig. 1B, Fig. S2A and Table S1). Splenic B and T cell populations in this mouse model were similar in numbers and characteristics to those of wild-type as assessed by cytofluorimetry (Fig. S2B). Moreover, analysis of CDR3s in primary BCR repertoires of these mice revealed an immensely diverse V_H_1-2-based CDR3 repertoire, as well as a diverse Vκ1-33 repertoire (Fig. 1, C and D and Table S2). Following immunization, we predicted that the massive CDR3 diversity would contribute to selection of B cells with human V_H_1-2- and Vκ1-33-based BCRs into germinal centers, where somatic hypermutations could further diversify variable region exons and contribute to affinity maturation.

### Identification of potent SARS-CoV-2 neutralizing antibodies

**Fig. 2.**
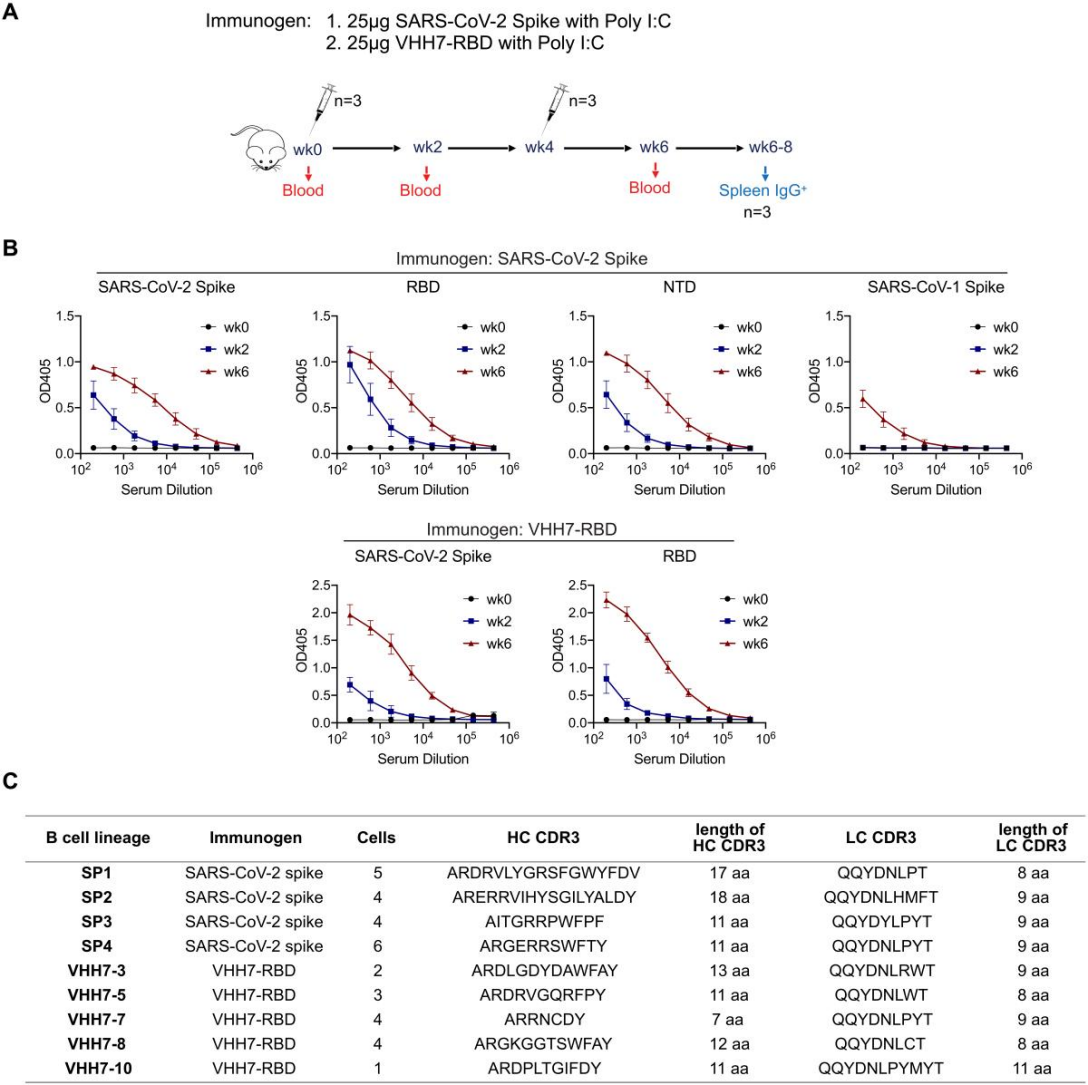
Immunizing the V_H_1-2/Vκ1-33-rearranging mouse model with SARS-CoV-2 spike or RBD elicited multiple V_H_1-2/Vκ1-33 antibodies. (**A**) Immunization scheme. Prime plus boost immunizations were performed at an interval of four weeks. (**B**) Binding curves showing reactivity of sera to SARS-CoV-2 spike, RBD, NTD and SARS-CoV-1 spike protein. The upper panel showed the sera from the SARS-CoV-2 Spike immunized mice at week 0, 2 and 6. The bottom panel showed the sera from VHH7-RBD immunized mice. Data were mean ± SD of three mice. (**C**) Table showed the V_H_1-2/Vκ1-33 antibodies isolated from SARS-CoV-2 spike-specific or RBD-specific IgG^+^ B cells. The antibody sequences and sequence features were shown in Table S3.

We immunized the human V_H_1-2/Vκ1-33-rearranging model with recombinant stabilized soluble SARS-CoV-2 Wuhan-Hu-1 spike trimer, or with an RBD monomer fused to a nanobody (“VHH7-RBD”) that targets MHC II-complex antigens (*38*) to potentially increase immunogenicity--given the poor immunogenicity of RBD monomer (*39*) (Fig. 2A). Immunizations were done twice, four weeks apart, with each immunogen plus poly I:C adjuvant (Fig. 2A). All mice developed strong antibody responses to the SARS-CoV-2 spike or RBD immunogens, as demonstrated by serum IgG titers at 2 weeks and 6 weeks post immunization (fig. 2B). At 3 weeks after the second immunization, 96 antigen-specific IgG^+^ B cells were fluorescence-activated single-cell sorted from each mouse and their IgH (HC) and IgL (LC) variable region exons were sequenced (Fig. S3A). These experiments identified 9 separate spike-specific B cell lineages, each of which expressed BCRs with unique sets of V_H_1-2 and Vκ1-33-associated CDR3s (Fig. 2C). In each CDR3-based lineage, individual members had unique patterns of somatic hypermutations (Table S3). The V_H_1-2-based HC CDR3s of these lineages were also distinct from CDR3s of previously reported V_H_1-2-based anti-SARS-CoV-2 antibodies. To assess binding properties, we expressed one monoclonal antibody from each lineage with human IgG1or IgG4 and human Igκ constant regions. ELISAs showed that all bound to the SARS-CoV-2 spike protein and 6 bound to the RBD, with one of the latter, termed SP1-77, derived from spike protein immunization (Fig. S3B).

**Fig. 3.**
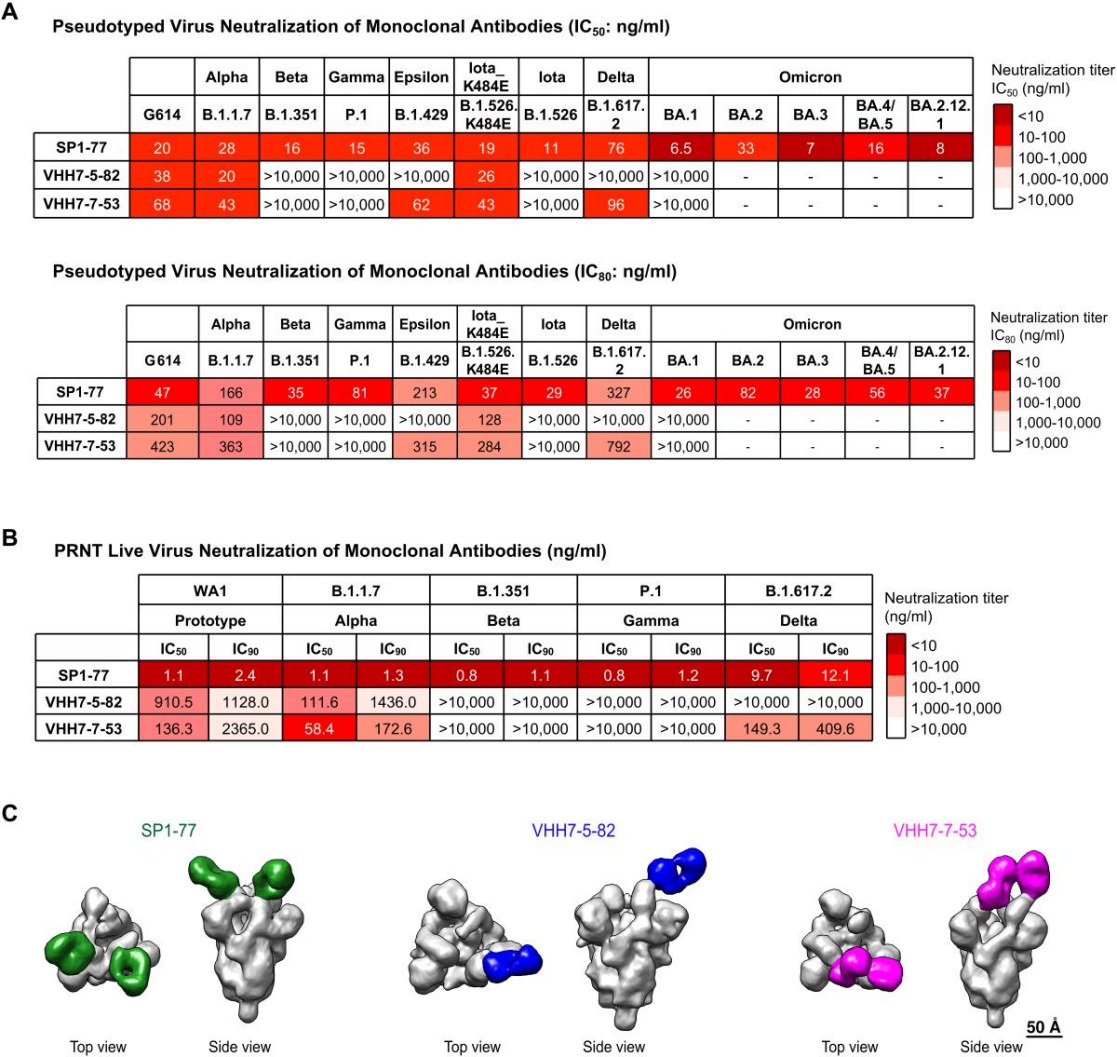
SP1-77 potently neutralized SARS-CoV-2 VOCs, including Omicron sub-variants. (**A**) Table showed the neutralization activities (IC_50_: top; IC_80_: bottom) of three monoclonal antibodies against all variants of concern (VOCs) and some variants of interest (VOIs) in pseudovirus neutralization assays. Experiments were done in 293 T/ACE2 cells. The neutralization curves were shown in Fig. S4A. The mutations on the spike proteins of different variants were listed in Table S4. Data were representative of 2 biologically independent experiments for most VOCs and VOIs, but one experiment for BA.3. Each independent experiment contained 2 technical replicates. IC_50_ and IC_80_ values were color-coded based on the key shown at the right. (**B**) Table showed the neutralization activities of three antibodies against VOCs in PRNT live virus neutralization assays. The neutralization curves were shown in fig. S4C. Data were representative of one independent experiment with 2 technical replicates. IC_50_ and IC_90_ values were color-coded based on the key shown at the right. (**C**) Final 3D reconstruction of Fab-S complexes shown in top view and side view with the S in gray and the Fabs colored (SP1-77: green; VHH7-5-82: blue; VHH7-7-53: magenta).

SARS-CoV-2 pseudovirus neutralization assays revealed that 3 of the RBD-binding monoclonal antibodies potently neutralized the SARS-CoV-2 G614 virus including VHH7-5-82 (IC_50_: 38 ng/ml), VHH7-7-53 (IC_50_: 68 ng/ml), and SP1-77 (IC_50_: 20 ng/ml) (Fig. 3A; Fig. S4A; Table S4). VHH7-5-82 also potently neutralized the Alpha (IC_50_: 20 ng/ml) and Iota (K484E) (IC_50_: 43 ng/ml) VOCs (Fig. 3A; Fig. S4A; Table S4). VHH7-7-53 potently neutralized Alpha (IC_50_: 43 ng/ml), Epsilon (IC_50_: 62 ng/ml), Iota (K484E) (IC_50_: 43 ng/ml) and Delta (IC_50_: 96 ng/ml) VOCs (Fig. 3A; Fig. S4A; Table S4). Notably, SP1-77 potently neutralized all currently known SARS-CoV-2 VOCs through BA.5, including robust neutralization of the recently emerged Omicron sub-variants, BA.1 (IC_50_: 6.5 ng/ml), BA.2 (IC_50_: 33 ng/ml), BA.3 (IC_50_: 7 ng/ml), BA.4/BA.5 (IC_50_: 16 ng/ml) and BA.2.12.1 (IC_50_: 8 ng/ml) (Fig. 3A; Fig. S4A; Table S4). The IC_80_ neutralization values of SP1-77 against these variants were equally compelling (Fig. 3A). Similar neutralization profiles for SP1-77 were found with an independent pseudovirus neutralization assay (Fig. S4B). Robust neutralization of all assayed variants (which did not include Omicron sub-variants) by SP1-77 also was seen in a plaque reduction neutralization test (PRNT) on live SARS-CoV-2 virus, with IC_50_ values ranging from 0.8 to 9.6 ng/ml (Fig. 3B and Fig. S4C). We also generated an SP1-77-derived antibody in which J_H_ and Jκ framework sequences outside of CDR3 were fully humanized and found that it retained similar robust neutralization activities against G614, Delta, and Omicron sub-variants as SP1-77 (Fig. S4D). Finally, we expressed the other four antibodies from the SP1 clonal lineage, each of which has unique pattern of somatic hypermutations compared to SP1-77. All had similar broad and potent neutralization activities (Fig. S4D).

Surface plasmon resonance (SPR) confirmed interactions of the Fabs of the 3 RBD-binding neutralizing antibodies with SARS-CoV-2 spike protein (Fig. S5A). We further imaged Spike-Fab complexes using negative-stain electron microscopy (NSEM) to elucidate binding characteristics (Fig. 3C and Fig. S5, B to D). Three-dimensional NSEM reconstructions indicated that VHH7-5-82, VHH7-7-53, and SP1-77 all bound to both up and down RBDs (Fig. 3C and Fig. S5, B to D). However, these reconstructions further indicated that these 3 antibodies had distinct binding footprints. Views from the top, front, inner, outer and back faces of the RBD showed that: VHH7-5-82 bound the top and outer faces of RBD, overlapping with the RBM (Fig. S6A); VHH7-7-53 bound the top face of the RBD, near the front, overlapping more widely with the RBM (Fig. S6B); and SP1-77 bound the back and outer faces, completely outside of the RBM (Fig. S6C). Indeed, a surface plasmon resonance competition assay showed that SP-77 did not compete with ACE2 for binding to the RBD or spike trimer (Fig. S6D). According to the Bjorkman nomenclature (*22*), VHH7-5-82 and SP1-77 were Class 2 and Class 3 antibodies, respectively, while VHH7-7-53 was somewhat intermediate between Class 1 or Class 2 antibodies. According to the Veesler nomenclature (*40*), SP1-77 bound site IV, VHH7-7-53 bound site Ib, and VHH7-5-82 bound somewhat intermediate between sites Ib and IV. Notably, 9 well-characterized V_H_1-2-based SARS-CoV-2 neutralizing antibodies isolated from SARS-CoV-2 Wuhan-Hu-1 or G614 virus-infected human patients all had very similar RBD binding footprints, which were distinct from those of VHH7-5-83, VHH7-7-53, and SP1-77 (*21*, *22*, *31*, *41*–*45*) (Fig. S7, A and B).

### cryo-EM structure of G614 S trimer in complex with the SP1-77 Fab

**Fig. 4.**
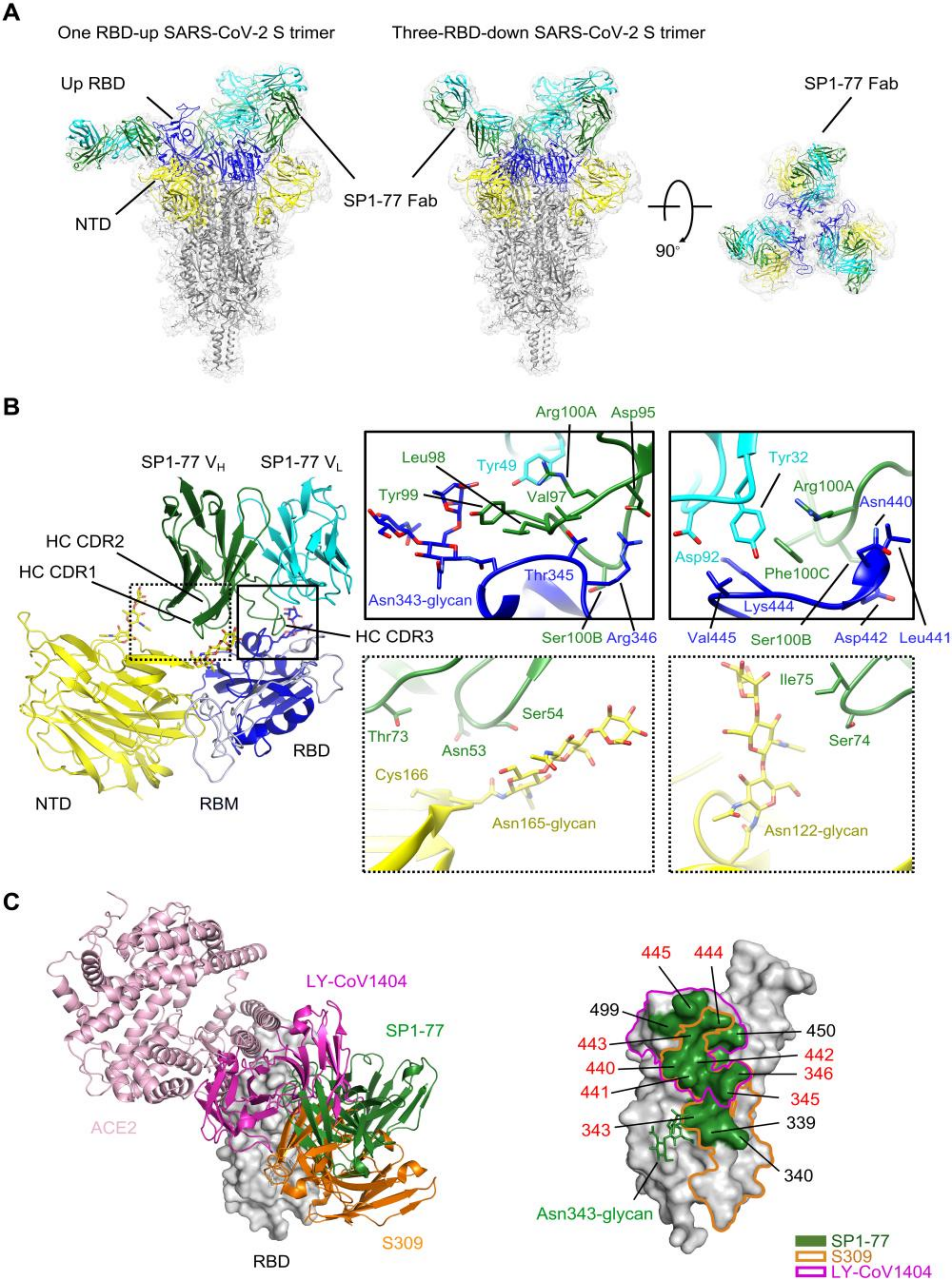
cryo-EM structures of SP1-77 Fab in complex of a full-length SASR-CoV-2 S trimer. (**A**) Cryo-EM structures of SP1-77 Fabs in complex with full-length S trimer in the one-RBD-up (2.9 Å) and three-RBD-down (2.7 Å) conformations. EM density was in gray and structures were in ribbon diagrams with RBD in blue, NTD in yellow and the rest in dark gray. SP1-77 HC was in green and LC was in cyan. Three SP1-77 Fabs bound one S trimer in both conformations. (**B**) Close-up view of interactions between SP1-77 Fab and the RBD and NTD of the S trimer in RBD-down conformation. Left, HC CDR3 of SP1-77 made main contact with RBD away from RBM in light blue, while HC CDR2 touched the N-linked glycan from the NTD. Right, zoom-in views of binding interface, showing 17-residue HC CDR3 wedging into a groove formed by two segments of residues 339-346 and residues 440-445. The Asn343 glycan of RBD also interacted with Tyr99 from HC CDR3. Two glycans at Asn122 and Asn165 in the NTD were in proximity to CDR2 (Asn53, Ser54, Gly56, Thr57 and Asn58) and HC FW3 (Thr73, Ser74 and Ile75). (**C**) Comparison of binding mode and footprint of SP1-77 with ACE2 and other neutralizing antibodies, including S309 and LY-CoV1404. Left, RBD was in surface representation in gray. ACE2 and the antibodies were in ribbon diagram with SP1-77 in green, S309 in orange, LY-CoV1404 in magenta, and ACE2 in pink. Right, footprints on RBD of various antibodies with SP1-77 outlined in green, S309 in orange and LY-CoV1404 in magenta. The interface residues of SP1-77 were indicated with major contacting residues in red.

As SP1-77 neutralized SARS-CoV-2 with remarkable potency and breadth, we determined cryo-EM structures of the full-length G614 S trimer in complex with the SP1-77 Fab (*46*) (Fig. S8) 3D classification revealed two distinct conformations, including a three-RBD-down conformation and a one-RBD-up conformation, that both bound to three Fabs per S trimer (Fig. 4A) -- consistent with negative stain EM results (Fig. S5D). These structures were refined to 2.7Å and 2.9Å resolution, respectively (Fig. S8 to S10 and Table S5). To improve resolution near the RBD/SP1-77 interface, we performed local refinement, leading to a 3.2Å map that covered the Fab-RBD interface from the S trimer in the three RBD-down conformation. From the S trimer in the one-RBD-up conformation, we obtained maps for the Fab-RBD interfaces in the down conformation and in the up conformation at 3.3Å and 4.8Å resolution, respectively (Fig. S8 to S10). These structures confirmed that the SP1-77 epitope sit on the opposite side of RBD from the ACE2 binding site (Fig. 4, B and C), consistent with the negative stain EM data and our surface plasmon resonance competition data showing that SP1-77 did not impact ACE2 binding.

SP1-77 mainly made direct contacts with the RBD, but also touched the N-terminal domain (NTD) from a neighboring protomer (Fig. 4B). The interaction buried an 939 Å^2^ surface area on the antibody and an 936 Å^2^ area from the spike protein. The HC and LC of SP1-77 contributed to 83% and 17% of the binding surface area, respectively. In particular, part of the 17-residue HC CDR3 wedged into a short groove formed by two segments in the RBD encompassing residues 343-346 and 441-444, respectively, while the tip of the 343-346 segment (mainly Thr345 and Arg346) projected into a ring-like structure formed by the HC CDR3 (Fig. 4B). The intertwined contacts between the HC CDR3 and the RBD accounted for 78% of the binding surface area contributed by the HC. There appeared to be an extensive hydrogen bond network between Asn343, Thr345, Arg346, Asn440, Leu441 from the RBD and Val97, Leu98, Tyr99, Gly100, Arg100A, Ser100B from the HC CDR3. Notably, there was also a salt bridge between Arg346 of the RBD and Asp95 of the HC CDR3. Tyr99 of the HC CDR3 also packed against the fucose residue from the fucosylated Asn-343 glycan (*47*), probably forming a CH-π interaction, which buried a surface area of ~150 Å^2^ (Fig. 4B). In addition, there were contacts between the 441-444 segment (Lys444 and Val445) and Tyr32 of the LC CDR1 and Asp92 of the LC CDR3 (Fig. 4B). Finally, the SP1-77 HC CDR2 and FW3 made additional contacts with the Asn122 and Asn165 glycans on the NTD, probably by forming hydrogen bonds or water-mediated hydrogen bonds (Fig. 4B). We noted that these two glycosylation sites were highly conserved among different VOCs (Fig. S11). Overall, the Cryo-EM structure revealed that, compared to other SARS-CoV-2 nentralizing antibodies, SP1-77 bound a different RBD epitope outside of the ACE2 binding motif via an interaction substantially mediated by the HC CDR3.

We compared the binding epitopes of SP1-77 with those of the LY-CoV1404 (Bebtelovimab) and S309 neutralizing antibodies. The RBD epitope targeted by SP1-77 overlapped very modestly with that of the LY-CoV1404, which is currently approved for therapeutic use (*14*) (Fig. 4C). The RBD-binding site of LY-CoV1404 included the 440-445 segment of the RBD that was also bound by SP1-77 (Fig. 4C). Like SP1-77, LY-CoV1404 potently neutralizes all currently known VOCs (*14*). However, the bulk of the LY-CoV1404 footprint overlaps with the RBM, which mechanistically allows LY-CoV1404 to achieve neutralization by blocking ACE2 binding. S309 and its Sotrovimab derivative, previously used therapeutically, bind outside of the RBM (*23*). However, S309 is quite distinct from SP1-77 in that it fails to neutralize Omicron BA.2 *in vitro* (*48*); although it does prevent BA.2 infection *in vivo* through utilization of Fc effector function interactions (*49*). In this regard, the S309 footprint covered both the 339-346 and 440-451 segments that were also in the SP1-77 footprint, with the long axis of the S309 footprint aligning along the exposed ridge of the RBD in the closed S trimer (Fig. 4C). Notably, however, the same axis of the SP1-77 footprint was almost perpendicular to that of S309, which made it possible for SP1-77 to reach the NTD (Fig. 4B).

### Comparison of SP1-77 footprint to the mutations on the RBD of variants.

**Fig. 5.**
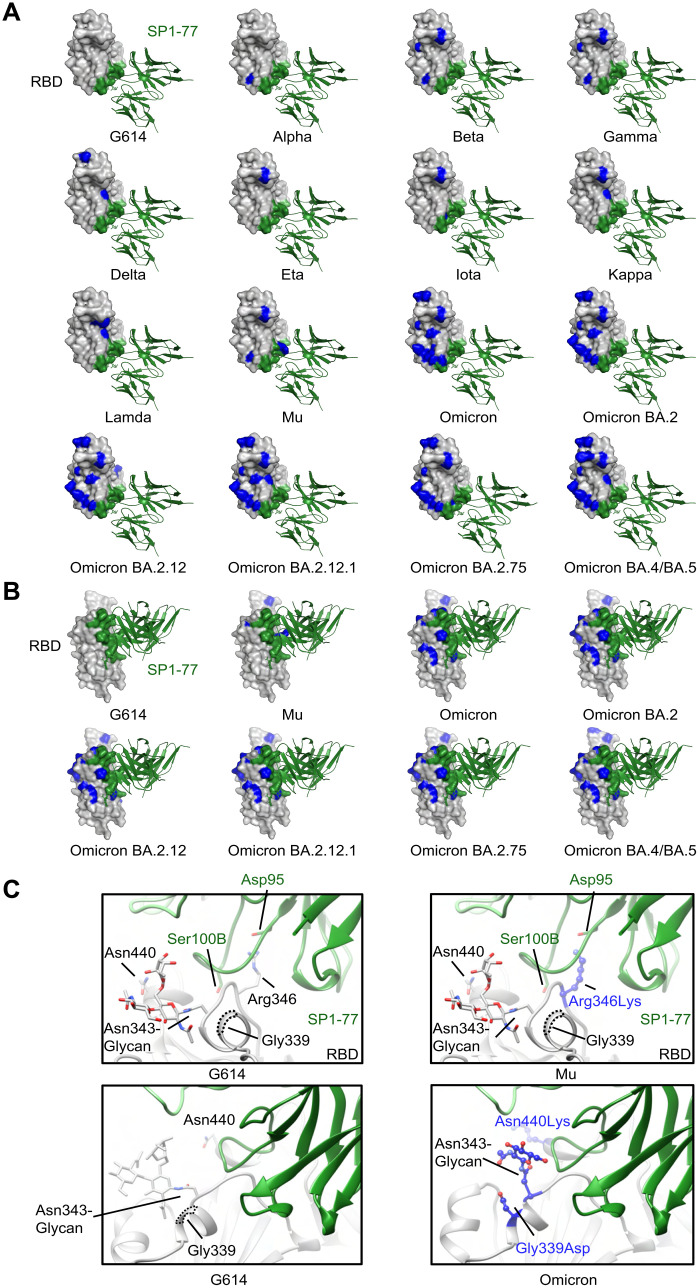
Modeled SP1-77 binding site on various SARS-CoV-2 variants. (**A**) The potential footprint of SP1-77 on the modeled RBDs from different SARS-CoV-2 variants in a top view. The RBD was shown in surface representation in gray with the SP1-77 footprint highlighted in green and the mutations in each variant in blue. The Fv region of SP1-77 was shown in ribbon diagram in green. Most mutations on spike variants were not located at the SP1-77 footprint. (**B**) Side view of a selected panel from **A**. (**C**) Structural comparison of the SP1-77 binding interface among the RBDs of wildtype G614, Mu and Omicron variants. The conservative mutation Arg346Lys in Mu preserved the salt bridge between the residue 346 in the RBD and the SP1-77 Asp95. The mutations in Omicron variant reconfigured the local conformation near the N343-glycan, which is on the edge of SP1-77 footprint. The RBD was colored in gray, the SP1-77 heavy chain was colored in green and the light chain was colored in light green. Mutations in the Mu and Omicron variants were shown in stick and ball model in blue.

When the SP1-77 footprint was projected onto modeled RBDs from different SARS-CoV-2 variants (Fig. 5, A and B), it was evident that most mutations were located outside of the SP1-77 binding site, except for the Arg346Lys in the Mu and Omicron-BA.1.1 variants, and the Gly339Asp and Asn440Lys in all current Omicron sub-variants (*50*). The modeled interfaces of SP1-77 bound to the Mu and Omicron RBDs, based on the SP1-77/G614 S complex, indicated that the conservative Arg346Lys mutation would preserve the interaction with HC CDR3 and would not have much impact on binding. Likewise, the Gly339Asp and Asn440Lys mutations were at the edge of the SP1-77 epitope with their side chains pointing away from the binding interface, explaining why Omicron variants were sensitive to SP1-77 neutralization (Fig. 5C). While mutations near the Asn343-glycan in Omicron reconfigure the orientation such that the carbohydrate projects away from the protein surface (*51*), such mutations had minimal impact on binding and neutralization activity of SP1-77 (Fig. 5C).Thus, these studies provided a structure-driven hypothesis for why SP1-77 potently and broadly neutralized all tested SARS-CoV-2 variants through Omicron BA.5. Finally, we compared the SP1-77 footprint with the new mutations on the recently emerged BA.2.75 sub-variant. The N460K mutation in BA.2.75 was far from the SP1-77 footprint (Fig. 5, A and B); while the BA.2.75 G339H and G446S mutations were close to but did not overlap with SP1-77 footprint as described for BA.1. These findings made us hopeful that SP1-77 also will bind and robustly neutralize BA.2.75.

### SP1-77 neutralized SAR2-CoV-2 by inhibiting viral-host membrane fusion.

**Fig. 6.**
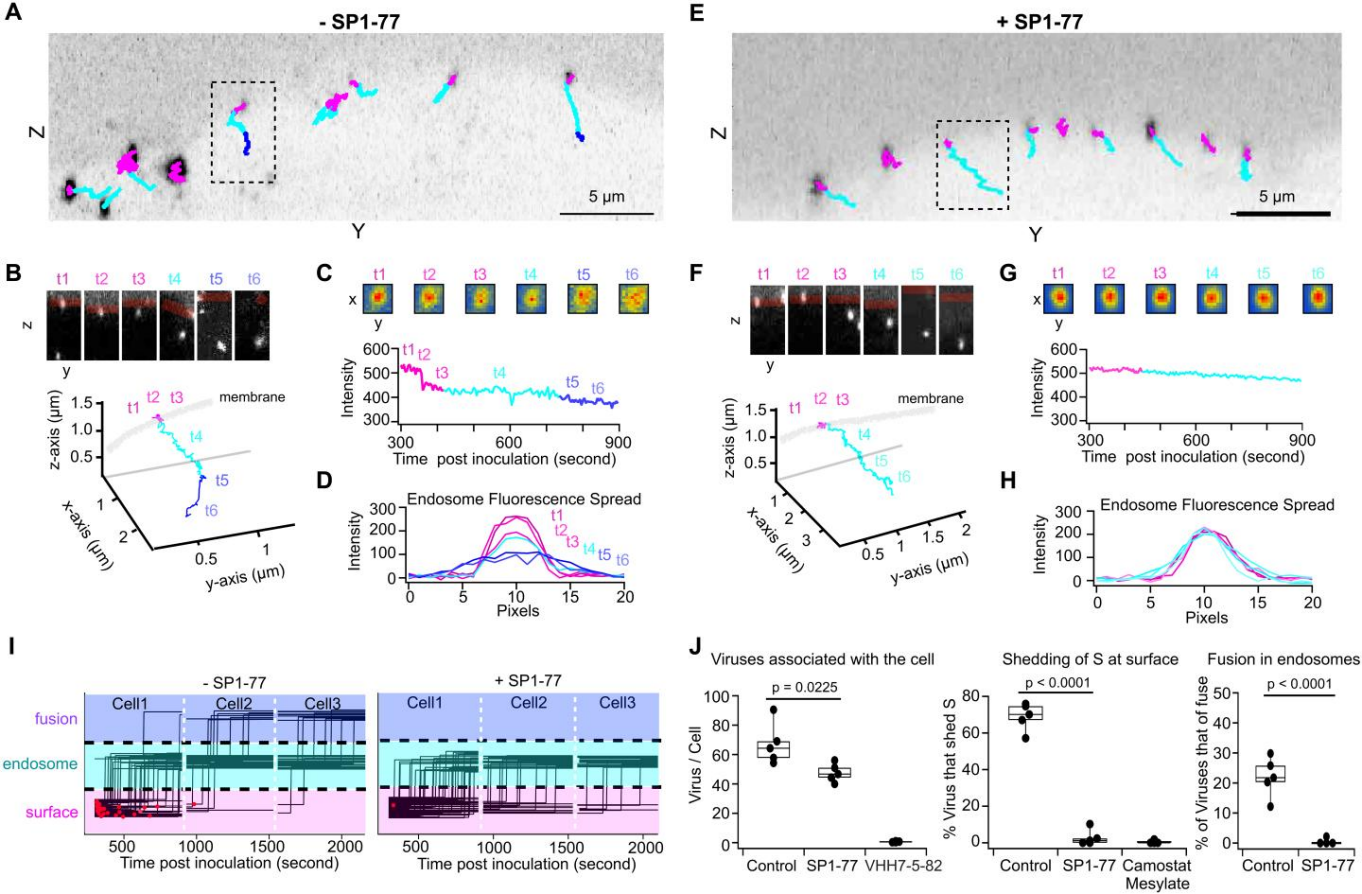
LLSM single virus tracking revealed SP1-77 inhibited S-fragment shedding and membrane fusion. (**A-I**) Trajectories of VSV-SARS-CoV-2-Atto 565 virus imaged every 4 seconds with volumetric LLSM beginning 5 minutes after inoculation of Vero TMPRSS2 cells with virus at MOI ~ 2 without (**A-D**) or with 500 ng/mL SP1-77 treatment (**E-H**). Trajectories were colored magenta when virus was localized to cell surface, light blue when virus internalized, and dark blue if the Atto 565 signal spreaded from a point spread function (PSF) to a larger distribution indicating viral envelope fusion with endosomal membrane. Single virus trajectories (**B, F**) with insets showing x-axis projection through 4 planes (top), 3D integrated intensity profiles (**C, G**, bottom) and corresponding heat maps of fluorescence intensities from 4 plane z- projections (**C, G**, top). Intensity line-profiles through center of virions **(D, H**) appeared as a single point spread function at the cell surface and in endosomes in the absence (**D**, t1-t4) or presence (**H**, t1-t6) of SP1-77. Atto 565 spreaded upon fusion in the absence of SP1-77 (**D**, t5-t6). (**I**) Summary of all single virus trajectories over the course of single experiments, in the absence and presence of SP1-77, each from 3 cells imaged consecutively for 10 min. Red dots indicated virus trajectories in which a TMPRSS2-dependent Atto 565 signal decrease was observed. (**J**) Quantification of 5 experiments for each condition for the number of viruses in the total cell volume of every cell imaged (left), the number of Atto 565 decreases observed in all the trajectories of virus when co-localizing to the cell surface (middle), and the number of instances of Atto 565 dye spreading within endosomes (right). Data were mean ± SD of 5 independent experiments. *p* values were accessed by unpaired two-tail t-test. Data were shown in Table S6.

To address the SP1-77 neutralization mechanism, we applied a lattice light sheet microscopy (LLSM) approach to monitor target cell infection by a chimeric vesicular stomatitis virus in which the coat glycoprotein was replaced by the SARS-CoV-2 (Wuhan strain) S protein (VSV-SARS-CoV-2) (Fig. 6 and Fig. S12) (*52*). This chimeric virus was surface labeled with Atto565-NHS-ester (VSV-SARS-CoV-2-Atto 565) (Fig. S12, A and B) and encoded a soluble eGFP that was expressed by infected cells. ACE2-expressing Vero cells that ectopically expressed TMPRSS2 protease (Vero TMPRSS2) were used as host cells. LLSM imaging revealed Atto 565-labeled viruses on cell surfaces at the earliest time points (Fig. 6, A to D, I, trajectories of viruses shown in magenta, light and dark blue). Single stepwise decreases in fluorescence intensity, corresponding to 25-30% of the total Atto 565 fluorescence, were observed for cell surface virus (Fig. 6, B to D, I, t2 to t3, magenta). As these stepwise decreases were blocked by pharmacological inhibition of TMPRSS2 (Camostat Mesylate) (Fig. 6J, middle panel), they likely resulted from TMPRSS2 protease activity and, correspondingly, reflected S1 dissociation after S2’ cleavage. In these experiments, viral-host membrane fusion was not observed on the cell surface as these experiments were done with culture medium at neutral pH, while membrane fusion requires acidic pH (*52*). A subset of labeled chimeric viruses were internalized via endocytosis within a recorded 10-min time series (Fig. 6, A to D, I, trajectories of viruses shown in light and dark blue), followed by fluorescence-spreading, indicative of membrane fusion within the endosome (Fig. 6, B to D, I, t5 to t6, dark blue; the method of single virus tracking and image analysis).

VSV-SARS-CoV-2-Atto 565 chimeric virus infectivity of Vero TMRSS2 cells was potently neutralized by its pre-incubation with SP1-77 IgG (IC_50_ ~ 15 ng/ml) (Fig. S12C). Pre-incubation of VSV-SARS-CoV-2 Atto 565 with 500 ng/ml of SP1-77 did not appreciably impact its binding to the Vero TMPRSS2 cell surface (Fig. 6, E to I, trajectories of viruses shown in magenta and light blue). This finding was consistent with the SP1-77 footprint lying outside of the RBM and the inability of SP1-77 to block ACE2 engagement (Fig. 4C, Fig. S6, C and D). In addition, pre-incubation with SP1-77 greatly diminished the number of Atto 565 fluorescence intensity step decreases of chimeric virus at the cell surface (Fig. 6, F to J, t2 to t3). This result indicated that SP1-77 may either inhibit S2’ cleavage or inhibit S1 dissociation (or inhibit both). While pre-incubation of viruses with SP1-77 had modest impact on virus endocytosis (Fig. 6, F to I, trajectories of viruses shown in light blue), it dramatically inhibited fluorescence-spreading within the endosome--indicative of a viral-endosomal membrane fusion block (Fig. 6, F to J). Together, these findings suggested that SP1-77 neutralized the SARS-CoV-2 by blocking membrane fusion (Fig. 6, I and J). For comparison, LLSM single virion visualization revealed that pre-incubation with 500 ng/ml of our VHH7-5-82 antibody fully blocked binding of the chimeric virus to the cell surface (Fig. 6J), consistent with our findings that the VHH7-5-82 footprint overlapped with the RBM (Fig. S6A).

### SP1-77 inhibited S1 dissocaition

**Fig. 7.**
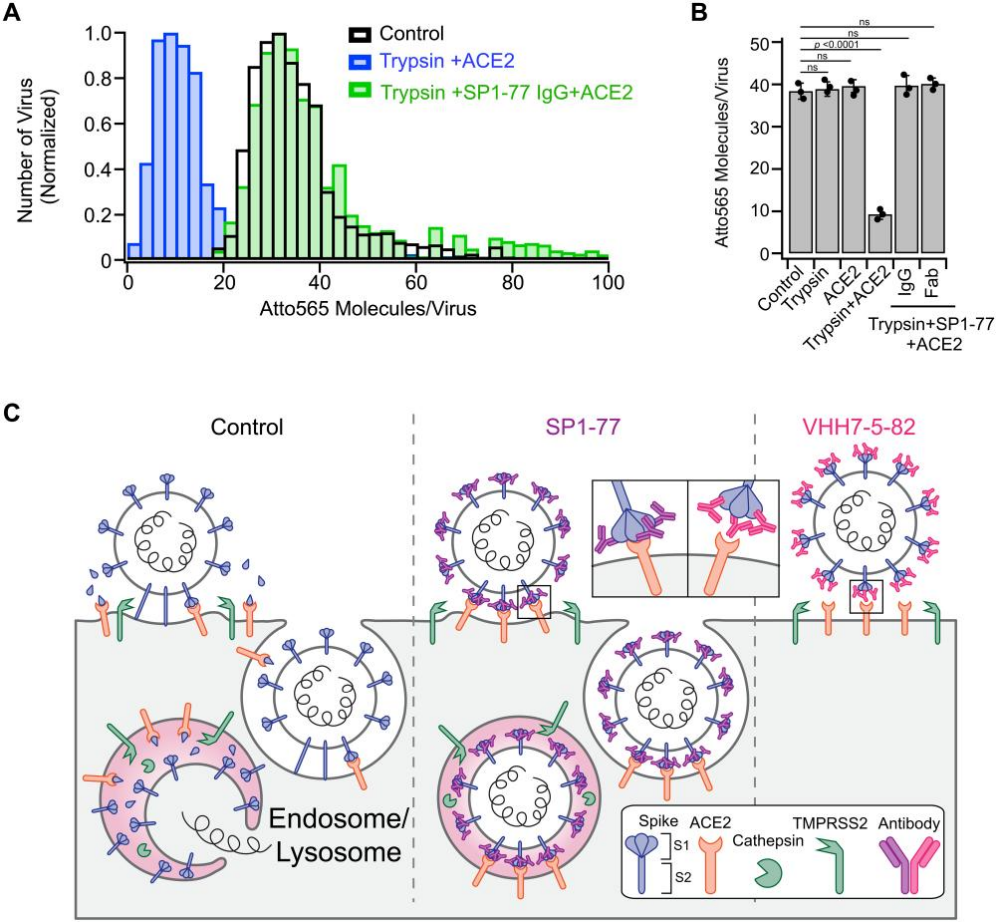
SP1-77 inhibited ACE2-mediated S1 dissociation on trypsin-treated VSV-SARS-CoV-2 Atto 565 viruses. (**A**) Histogram of the number of Atto 565 molecules on VSV-SARS-CoV-2-Atto 565 determined by single molecule counting. (**B**) Histogram of averages of peak distribution determined by a Gaussian fit of 3 independent experiments. *p* values were accessed by unpaired two-tail t-test. Data were shown in Table S7. Treatments prior to adsorption included none (control), incubation with 1 μg/mL trypsin for 30 min at 37°C, treatment without or with trypsin followed by an incubation with 0.5 μM of recombinant ACE2 for 10 min at 37°C, or treatment with trypsin then incubated with 100 ng/mL SP1-77 IgG or SP1-77 Fab for 1 hour at 37°C followed by incubation with ACE2. (**C**) Schematic representation of proposed mechanism of SP1-77 inhibition of SARS-CoV-2 infection. Left panel: Without antibody treatments, the spike protein on the viral surface bound to the ACE2 receptor on the infected cell surface. Membrane fusion was activated either by TMPRSS2 protease on the cell membrane or by cathepsin L protease following endocytosis. Cleavage at the S2’ site by these proteases led to dissociation of the S1 subunit, which exposed the fusion peptide on the S2, facilitating viral-host membrane fusion and viral entry into the infected cells. Middle panel: Pre-treatment of the virus with SP1-77, a non-ACE2-blocking antibody, did not appreciably impact binding of viruses to the cell surface and their endocytosis. However, SP1-77 greatly inhibited the dissociation of S1 subunit, thereby, blocking activation of the fusion peptide and membrane fusion. Right panel: Pre-treatment of the virus with VHH7-5-82, an ACE2-blocking antibody, prevented binding of the virus to the cell surface.

To distinguish between the possible effect of SP1-77 on cleavage of S2’ versus dissociation of S1, we reconstructed these two processes *in vitro*. Activation of VSV-SARS-CoV-2-Atto 565 by brief treatment with trypsin for 30 minutes followed by the addition of the trypsin inhibitor aprotinin bypassed the requirement for host cell proteases TMPRSS2 and cathepsin L for infection--implicating that trypsin treatment cleaved the S2’ site (*52*, *53*). Incubation of the trypsin-activated virus with recombinant ACE2 induced a signal decrease of ~75% of the total Atto565 fluorescence, as quantified by single particle imaging; while cleavage by trypsin alone or incubation with ACE2 alone did not induce this signal decrease (Fig. 7, A and B). Assuming that an ~75% signal decrease *in vitro* reflected complete S1 dissociation from VSV-SARS-CoV-2 virus, then the corresponding 25-30 % signal decrease in vivo likely reflected S1 dissociation for a subset of spike proteins when the virus was attached to the cell surface (Fig. 6, B to D, t2 to t3). In contrast, the decrease in signal no longer occurred when trypsin-activated VSV-SARS-CoV-2-Atto 565 was incubated with SP1-77 and then exposed to ACE2 (Fig. 7, A and B). This finding indicated that SP1-77 inhibited ACE2-induced dissociation of S1 from the pre-cleaved S1/S2 complex. Finally, the SP1-77 Fab also inhibited S1 dissociation in this assay and, correspondingly, robustly neutralized VSV-SARS-CoV-2 virus (Fig. 7B andand Fig. S12C). Together, these findings indicated that SP1-77 can achieve neutralization activity by blocking S1 dissociation via a monovalent interaction (Fig. 7C).

## DISCUSSION

We have engineered the human V_H_1-2/Vκ1-33-rearranging mouse line that produced bnnnnnnnnnnopdiverse BCR repertoires through V(D)J recombination of these single human V_H_ and Vκ sequences in association with diverse CDR3 sequences generated by V(D)J junctional diversification mechanisms. Immunization of this mouse line with SARS-CoV-2 (Wuhan-Hu-1) spike protein immunogens elicited several humanized V_H_1-2/Vκ1-33-based SARS-CoV-2-neutralizing antibodies that bound RBD in a different mode from each other and from those of many prior human patient-derived V_H_1-2-based neutralizing antibodies. Of these three antibodies, two (VHH7-5-82 and VHH7-7-53), like many other previously described SARS-CoV-2 neutralizing antibodies, bound an epitope overlapping with the RBM. In this regard, our LLSM-based mechanism of action study directly demonstrated that VHH7-5-82 neutralized SARS-CoV-2 by blocking its binding to the host cell. The third antibody, SP1-77, bound RBD on the opposite side from the RBM. SP1-77 showed much broader neutralizing activity than the other two, and potently neutralized all major SARS-CoV-2 variants through the recently emergent BA.5 variant. Cryo-EM studies revealed that SP1-77 binding epitope on the SARS-CoV-2 RBD was different from those of previously characterized neutralizing antibodies. However, future mutagenesis studies are needed to more precisely define residues that are critical for SP1-77 binding to the RBD. The LLSM assay further revealed that SP1-77 did not inhibit SARS-CoV-2 binding to the host cell or its endocytosis. Instead, SP1-77 inhibited a downstream step in the SAR-CoV-2 infection process that involved fusion of the virus membrane with that of the host cell.

The SP1-77 neutralization mechanism we described provided further insight into how a non-ACE2 blocking antibody can potently neutralize SARS-CoV-2. Besides the S309 neutralizing antibody mentiond above, several other Omicron sub-variant neutralizing antibodies that have binding epitopes outside of the RBM have been reported (*1*, *54*). However, the mechanisms by which these antibodies neutralize SARS-CoV-2 remain unknown. The LLSM-based approach we employed to elucidate the SP1-77 mechanism should be readily applicable for mechanistic studies of other neutralizing antibodies that do not function via an ACE2 blocking mechanism. In the current study, the broad and potent activity of SP1-77 in neutralization of SARS-CoV-2 has only been demonstrated via pseudovirus assays. To further demonstrate the potential therapeutic utility of the antibody, we will need to perform live virus neutralization of omicron. Then, if the broad and potent SP1-77 neutralization activity against SARS-CoV-2 variants is maintained *in vivo,* it would have potential to be a therapeutic against current and newly-arising VOCs. Also, SP1-77 might be useful in a cocktail with other neutralizing antibodies, such as LY-CoV1404, that potently neutralizes all tested VOCs through an ACE2-blocking mechanism. In addition, the non-traditional neutralization mechanism of SP1-77 may inspire the design of new vaccine strategies to more broadly target BA.5 and other VOCs.

The use of a solely human V_H_1-2-rearranging mouse to obtain SP1-77 posed a rigorous test for this for the ability of such a mouse model to provide potent SARS-CoV-2-neutralizing humanized antibodies. As described above, full characterized V_H_1-2-based SARS-CoV-2 neutralizing antibodies isolated from Wuhan-Hu-1 or G614 SARS-CoV-2 virus-infected human patients bind a similar RBD epitope (*21*, *22*, *31*, *41*-*45*). None of these antibodies neutralize Beta, Gamma or Omicron variants, due to their sensitivity to the E484K/A mutation in these variants (*21*, *22*, *31*, *41*–*45*). Indeed, the striking binding and neutralization similarities of all patient-derived VH1-2-based neutralizing antibodies characterized in depth suggests that most VH1-2-based SARS-CoV-2-neutralizing antibodies in infected humans are selected by a common prototype neutralization pathway mediated by predisposition of the germline-encoded VH1-2 variable region to bind to the RBD (*31*). Notably, the footprints of our 3 V_H_1-2-based SARS-CoV-2 neutralizing antibodies obtained from the initial immunization of the human V_H_1-2/Vκ1-33-rearranging mouse were distinct from those of V_H_1-2 based SARS-CoV-2 neutralizing antibodies isolated from human patients. This finding highlights the ability of V_H_1-2/Vκ1-33-rearranging type of mouse model to permit isolation of potent human V_H_1-2-based SARS-CoV-2 neutralizing antibodies with different binding characteristics relative to those, thus far, derived from patients.

The V_H_1-2/Vκ1-33-rearranging mouse model has served as a very successful prototype for documenting the potential utility of this humanized antibody-discovery approach. In this regard, this intial study provided the VHH7-7-53 humanized antibody that potently neutralized multiple SARS-CoV-2 variants including Delta and the humanized SP1-77 antibody that potently neutralized all SARS-CoV-2 variants tested, including all Omicron sub-variants through BA.5. In this regard, among the thousands of SARS-CoV-2 neutralizing antibodies isolated from hundreds of human patients and vaccinated individuals, only a few broadly and potently neutralize all SARS-CoV-2 variants (*1*, *13*–*15*).

Our current study provided proof-of-principal that our mouse model could be used to identify humanized antibodies with potent activity against SARS-CoV-2 variants. However, we have only demonstrated the utility of mouse model for one human V_H_ and V_L_ combination and identified only just one potent and broad SARS-CoV-2 neutralizing humanized antibody. To demonstrate the broader utility of the approach, mouse models that incorporate additional V_H_s and V_L_s should be generated and tested. Ultimately, this type of mouse model may be useful for generating humanized antibodies more generally, including antbodies against other diverse pathogens for which rare cross-reactive neutralizing antibodies are needed such as HIV-1 and influenza.

## MATERIALS AND METHODS

### Study Design

The main objective of this study was to develop a humanized mouse model that through appropriate immunizations could be used to generate antibodes that broadly and robustly neutralize SARS-CoV-2 VOCs. The specific hypothesis tested was that such mice could be generated by programming them to produce sufficiently diverse antibody precursor repertoires through diversification mechanisms associated with the rearrangment of just a single human antibody heavy and light chain variable region sequence out of the 100 s present in the human genome. We generated such mice through standard gene targeted mutational *Igh* and *Igκ* locus modifications that exploited our recent discovery of genetic elements that could be modified to enhance the rearrangment and diversification of single human variable region sequences. We employed high throughput antibody repertoire analyses developed in our lab to document production in these mice of highly diverse antibody repertories from single human antibody variable region sequences via mechanisms that somatically diversify V(D)J rearrangements. We then immunized the mice with SARS-CoV-2 spike protein constructs and analyzed their antibody responses. We found that the mice indeed could make humanized antibodies that potently neutralized SARS-CoV-2. We focused on characterizing one of these antibodies that broadly and potently neutralized all major SARS-CoV-2 variants at least through the recently emergent BA.5 variant. We performed Cryo-EM studies that revealed that this antibody binds to an overall epitope on the SARS-CoV-2 Spike protein receptor binding domain that was different than any found to date. We further used a lattice-light-sheet-microsopy-based assay to reveal that this antibody inhibits a downstream step in the SARS-CoV-2 infection process that involves fusion of the virus membrane with that of the host cell.

### V_H_1-2/Vκ1-33-rearranging mouse model and embryonic stem cells

All the genetic modifications were introduced into previously generated V_H_1-2^IGCR1∆^ ES cells (129/Sv and C57BL/6 F1 hybrid background) (*33*), using targeting strategies described previously (*33*, *55*). In the *Igh* locus, the deletion of all mouse V_H_s was mediated by two guide RNAs that, respectively, targeted the upstream of the most distal mouse V_H_1-86P and the upstream of the human V_H_1-2 that replaced V_H_5-2 in this modified ES cell line. In the *Igκ* locus, the mouse Vκ3-2 segment was replaced with human Vκ1-33 segment with an attached CTCF-binding element (CBE) inserted 50 bp downstream of human Vκ1-33 segment. The replacement was mediated by homologous recombination using a PGKneolox2DTA.2 (Addgene #13449) construct and two guide RNAs that targeted the mouse Vκ3-2 segment. The Cer/sis regulatory element in this ES cell line was deleted via the use of two guide RNAs. A human TdT gene was expressed in this mouse model. The human TdT cDNA was inserted into the first intron of mouse Rosa26 gene by homologous recombination using a CTV (Addgene #15912) construct. The sequences of guide RNAs used for targeting were listed in table S9.

The V_H_1-2^mVH∆/IGCR1∆^/Vκ1-33^CS∆^ -rearranging mouse was generated by blastocyst injection of the ES cells described above and several rounds of breeding to get germline transmission and homozygous mice. All mouse experiments were performed under protocol 20-08-4242R approved by the Institutional Animal Care and Use Committee of Boston Children’s Hospital. More details on this protocol can be found in the supplementary materials.

### Splenic B Cell Purification and HTGTS-Repertoire-seq Analysis

Splenic B cells were purified from 5-8 week old mice by MACS Microbeads according to the manufacturer’s protocol. 10 ug of genomic DNA from purified splenic B cells was used for generating HTGTS-repertoire-seq (HTGTS-rep-seq) libraries as previously described (*56*). Four bait primers that target mouse J_H_1, J_H_2, J_H_3 and J_H_4 were mixed to capture the *Igh* heavy chain repertoire in one library. Likewise, four 4 bait primers that target mouse Jκ1, Jκ2, Jκ4 and Jκ5 were mixed to capture the *Igκ* light chain repertoire in one library. The sequences of mouse J_H_ and Jκ primers were as same as the previously reported (*56*). These HTGTS-rep-seq libraries were sequenced by Illumina NextSeq 2 x 150-bp paired end kit and analyzed with the HTGTS-Rep-seq pipeline (*57*).

### Immunogens and Immunizations

HexaPro Spike immunogen was produced and purified as previously described (*58*). HexaPro Spike was stabilized by the introduction of six prolines in the S2 region and contained an HRV 3C-cleavable C-terminal twinStrepTagII-8 × His tag to facilitate purification (PMID: 32703906). The VHH7-RBD immunogen was made as previously described (*38*). The VHH7-RBD immunogen is a recombinant protein that consists of a fusion between VHH7 nanobody which targets class II MHC antigens and the SARS-CoV-2 RBD (Wuhan Hu-1 strain). For each immunization, 200 ul of immunogen mix, containing 25 ug of filter-sterilized protein immunogen and 60 ug of Poly I:C in PBS, was injected into the peritoneum of each mouse. 8 weeks old mice were immunized twice with same immunogen, 4 weeks apart. More details on this protocol can be found in the supplementary materials.

### Antigen-specific B Cell Sorting and Single Cell RT-PCR

Mouse spleen samples from immunized mice were processed for single B cell sorting as previously described (*56*). Antigen-specific IgG^+^ B cells were selected for the phenotype B220^+^ (PE-Cy7: BD 552772), IgD^−^ (BV711: BioLegend 103255), IgG1^+^ (BV605: BD 563285), IgG2a^+^ (BV605: BD 564024), Spike^+^ (Alex647) or RBD^+^ (Alex647). SARS-CoV-2 spike and RBD were conjugated with Alex647 fluorescence (Thermo Fisher A30009). Single cell RT-PCR was performed as described previously (*33*). In brief, single antigen-specific IgG^+^ B cell was sorted into 96-well plate. Single cell cDNA was generated by reverse transcription (Thermo Fisher 18080093) using a primer mixture that specifically target Cu, Cγ1, Cγ2a and Cκ. V_H_1-2 and Vκ1-33 V(D)J exons were amplified by two rounds of nested PCR and identified by sanger sequencing. Primer sequences used are listed in table S9.

### Monoclonal Antibody and Fab Production

The antibody expression constructs containing the heavy-chain and the light-chain variable region exons (table S3), with human constant region sequences (IgG4 or IgG1, Igκ) at the C terminus were made by Genscript. Monoclonal antibodies were generated using the Expi293 expression system (Thermo Fisher Scientific) and purified by high-performance liquid chromatography (HPLC) coupled with HiTrap Protein A HP columns (Cytiva). Fabs were made as previously described (*59*). Full-length antibodies at ~20 mg/ml concentrate were digested by papain-agarose resin. Then, the Fab fragments were collected by rProtein A Sepharose Fast Flow (Cytiva).

### ELISA

ELISA was preformed as previously described (*60*). In brief, 96 well plates (Thermo Fisher 446612) were coated with SARS-CoV-2 spike, RBD, NTD or SARS-CoV-1 spike at 100 ng/well. Sera were added to the plates in the dilution series from 1:200. After washing, antibodies retained on the plates were detected with AP anti-mouse IgG antibody (Southern Biotech, 1030-04).

### Pseudovirus Neutralization Tests

The pseudovirus neutralization assay in 293 T/ACE2 cells was performed at Duke (*61*). The mutations on the spike protein of SARS-CoV-2 variants were listed in table S4. Spike-pseudovirus was incubated with 8 serial 5-fold dilutions of antibody in duplicate for 1 hr. at 37°C in 96-well culture plates. 293 T/ACE2-MF cells were detached from culture flasks, suspended in growth medium (GM), and immediately added to all wells. One set of 8 wells received cells + virus (virus control) and another set of 8 wells received cells only (background control). After 71-73 hrs of incubation, medium was removed and Promega 1X lysis buffer was added to all wells. After a 10-minute room temperature incubation, Bright-Glo luciferase reagent was added to all wells. After 1-2 minutes, the cell lysate was transferred to a black/white plate. Luminescence was measured using a GloMax Navigator luminometer (Promega). Neutralization titers were the inhibitory concentration (IC) of antibody at which relative luminescence units were reduced by 50% (IC_50_) compared to virus control wells. More details on this protocol can be found in the supplementary materials.

### Plaque Reduction Neutralization Test (PRNT)

SARS-CoV-2 Plaque Reduction Neutralization Test (PRNT) were performed in the Duke Regional Biocontaiment Laboratory BSL3 (Durham, NC) as previously described with virus-specific modifications (*62*). Briefly, two-fold dilutions of antibody (the start concentration is 10 ug/ml) were incubated with 50 PFU SARS-CoV-2 virus (WA1, Alpha, Beta, Gamma and Delta) for 1 hour. The antibody/virus mixture was used to inoculate Vero E6 cells in a standard plaque assay (*63*, *64*). Briefly, infected cultures were incubated at 37°C, 5% CO2 for 1 hour. At the end of the incubation, 1 mL of a viscous overlay (1:1 2X DMEM and 1.2% methylcellulose) was added to each well. Plates were incubated for 4 days. After fixation, staining and washing, plates were dried and plaques from each dilution of each sample were counted. Data were reported as the concentration at which 50% of input virus was neutralized. A known neutralizing control antibody was included in each batch run (Clone D001; SINO, CAT# 40150-D001).

### Antibody Affinity and Competition Assays by Surface Plasmon Resonance (SPR)

SPR measurements of monoclonal Fabs binding to SARS-CoV-2 spike (S) proteins were performed using a Biacore S200 instrument (Cytiva) and as described previously (*58*). Antibody binding competition and blocking were measured by SPR using methods described previously (*58*). More details on this protocol can be found in the supplementary materials.

### NSEM Methods

Fab-spike complexes were negatively stained with 2 g/dL uranyl formate, imaged on a Philips EM420 electron microscope; and 3D reconstructions were calculated using standard protocols with Relion 3.0. More details on this protocol can be found in the supplementary materials.

### Cryo-EM single particle analysis

Cry-EM single particle analysis was carried out following the detailed protocols published previously (*65*, *66*). Briefly, to prepare cryo EM grids, the full-length G614 spike trimer (3.1 mg/ml) (*65*) and the SP1-77 Fab (7 mg/ml) were mixed at a molar ratio of 1:9 and the complex was also purified by gel filtration chromatography. The grids were first screened for ice thickness and particle distribution. Selected grids were used to acquire images by a Titan Krios transmission electron microscope (ThermoFisher Scientific) operated at 300 keV and equipped with a BioQuantum GIF/K3 direct electron detector. Automated data collection was carried out using SerialEM version 3.8.6 (*67*) with a defocus range of 0.5-2.2 μm.

Cryo-EM images were processed in cryoSPARC v.3.3.1. (*46*) with the drift correction performed and contrast transfer function (CTF) estimated. Motion corrected sums with dose-weighting were used for subsequent image processing. Template-based particle picking was performed for all 28,899 recorded images with 7,657,522 particles extracted in total. The particles were subjected to multiple rounds of 2D and 3D classification, giving two major classes, one representing the three-RBD-down S trimer bound with three SP1-77 Fabs and another representing the one-RBD-up S trimer in complex with three SP1-77 Fabs. The two classes class were refined to 2.9 Å resolution from 250,319 particles and 3.1 Å resolution from 209,249 particles, respectively. To further improve the density for the Fab and RBD, different local refinements were performed with a soft mask covering a single RBD and the bound Fab were performed. All resolutions were reported from the gold-standard Fourier shell correlation (FSC) using the 0.143 criterion and local resolution determined using cryoSPARC. For model building, we used our G614 S trimer structures (PDB ID: 7KRQ and PDB ID: 7KRR (*66*)) as the initial templates for the spike protein, and the S309 antibody structure (PDB ID: 6WS6 (*23*)) for SP1-77. Several rounds of manual building were performed in Coot (*68*), and iterative refinement was performed in both Phenix (*69*) and ISOLDE (*70*). The refinement statistics were summarized in table S5. More details on this protocol can be found in the supplementary materials.

### Generation of VSV-SARS-CoV-2 Atto 565

The generation of recombinant VSV chimeras expressing eGFP where the glycoprotein G was replaced with spike (S) protein Wuhan-Hu-1 strain (VSV-SARS-CoV-2) was previously described (*71*). VSV-SARS-CoV-2 at a concentration of ~150 μg/mL viral RNA was conjugated with Atto565-NHS ester (Sigma-Aldrich, cat.72464) (*52*). The labeled virus was first tested for its infection efficacy in Vero-TMPRSS2 cells as previously described (*71*). Then, 1-hour incubation of label virus with 100 ng/ml SP1-77 IgG or SP1-77 Fab before infection was performed to test the infection inhibition efficacy (neutralization activity) of SP1-77 IgG or SP1-77 Fab. More details on this protocol can be found in the supplementary materials

### Lattice light sheet microscopy (LLSM)

The single virus tracking LLSM experiments were performed as previous described (*52*). In brief, cells were plated onto 5 mm coverslips in a 35 mm culture dish at 60% confluence the day prior to each experiment. Approximately 10 μl of a solution containing 2.5 μg/mL viral RNA of VSV-SARS-CoV-2 Atto 565 (MOI ~2) without antibody (control), with 500 ng/ml SP1-77 or with VHH7-5-82 antibodies were incubated at at 37°C for 1 hour and then added to the coverslips. The cells were imaged in phenol red free media (FluoBright) supplemented with 5% FBS and 25 mM HEPES, pH 7.4. The samples were volumetrically imaged as a time series using a dithered multi-Bessel lattice light sheet using a sample scan with 0.5 μm spacing between each plane every 4 seconds.

### Single virus tracking and image analysis

The single virus tracking and image analysis was performed as previously described (*52*). The 3D stacks from the LLSM experiments were deskewed and the diffraction limited spots were detected and tracked using an automated detection algorithm that uses least-squares numerical fitting with a model of the microscope PSF approximated by a 3D Gaussian function and implemented in MATLAB previously developed (*72*). Estimated fluorescence intensities associated with each spot were calculated from the corresponding amplitudes of the fitted 3D Gaussian and compared to single virus bound to poly-D-lysine coated glass imaged under the same condition as the experiments. Tracks of virus were then exported into a custom-made program written in LabView for visualizing trajectories, which was available for download (https://github.com/VolkerKirchheim/TrackBrowser_Matlab.git, courteously provided by Volker Kiessling at the University of Virginia). Each virus trajectory was visually examined for localization to the cell surface, internalization into the cell, and spreading of the Atto565 dye fluorescence. The virus is 80-100 nm, which made it a diffraction limited object. The spot limited by the wavelength of the dye being emitted was 300 nm. The endosome was 600 nm and larger. The fusion of the viral envelope to the endosomal membrane was observed by a spreading of the fluorescent signal from a point spread limited object to 500-1000 nm over the endosomal membrane.

### S1 dissociation reconstitution

The S1 dissociation reconstitution was performed as previously described (*52*). In brief, VSV-SARS-CoV-2 Atto 565 was treated with 5 different conditions: trypsin only, ACE2 only, trypsin followed by ACE2, trypsin followed by SP1-77 IgG followed by ACE2 and trypsin followed by SP1-77 Fab followed by ACE2. The trypsin treatment was performed by incubation of VSV-SARS-CoV-2 Atto 565 with 1 μg/mL trypsin for 30 minutes at 37°C, and then the reaction was stopped by the addition of Aprotinin to a final concentration 10 μM. The ACE2 treatment was performed by incubation of VSV-SARS-CoV-2 Atto 565 with soluble ACE2 for 10 mininus at 37°C. The antibody treatment was performed by incubation of VSV-SARS-CoV-2 Atto 565 with 100 ng/ml IgG or Fab for 1 hour at 37°C. After treatment, virus was bound to poly-D-lysine-coated glass and single molecule Atto565 dye calibration was performed as previously described (*52*).

### Statistics

Statistical analyses in this paper were performed in Prism (v.8, GraphPad Software). *p* values were calculated by unpaired, two-tail t-test.

## Supplementary Materials

### This PDF file includes:

Materials and Methods Figs. S1 to S12 References (*73*–*99*)

## Notes

View/request a protocol for this paper from *Bio-protocol*.
