# Supplementary material for: An Antibody from Single Human VH-rearranging Mouse Neutralizes All SARS-CoV-2 Variants Through BA.5 by Inhibiting Membrane Fusion
Source: Sci Immunol. 2022 Aug 11:eadd5446. doi: 10.1126/sciimmunol.add5446 (PMC9407951; doi:10.1126/sciimmunol.add5446)
Supplement: Supplementary file 1 — Materials and Methods Figs. S1 to S12 References (73–99) [file sciimmunol.add5446_sm.pdf]

Science Immunology
AAAS

Supplementary Materials for

An Antibody from Single Human VH-rearranging Mouse Neutralizes All SARS-CoV-2 Variants Through BA.5 by Inhibiting Membrane Fusion

Sai Luo *et al.*

Corresponding author: Sai Luo, sai.luo@childrens.harvard.edu; Tom Kirchhausen, kirchhau@crystal.harvard.edu; Bing Chen, bchen@crystal.harvard.edu; Barton F. Haynes, barton.haynes@duke.edu; Frederick W. Alt, alt@enders.tch.harvard.edu

Sci. Immunol. **7**, eadd5446 (2022)
DOI: 10.1126/sciimmunol.add5446

The PDF file includes:

Materials and Methods
Figs. S1 to S12
References (*73–99*)

Other Supplementary Material for this manuscript includes the following:

Tables S1 to S9

Material and methods

V_H1-2/Vκ1-33-rearranging mouse model and embryonic stem cells

All the genetic modifications were introduced into previously generated $V_H1\text{-}2^{IGCR1\Delta}$ ES cells (129/Sv and C57BL/6 F1 hybrid background) (*73*), using targeting strategies described previously (*73, 74*). In the *Igh* locus, the deletion of all mouse V_Hs was mediated by two guide RNAs that, respectively, target sequences ~100 bp upstream of the most distal mouse V_H1-86P and ~4.3 kb upstream of the human V_H1-2 that replaced V_H5-2 in this modified ES cell line, respectively. In the *Igκ* locus, the mouse Vκ3-2 segment was replaced with human Vκ1-33 segment with an attached CTCF-binding element (CBE) (atccaggaccagcagggggcgcggagagcacaca) inserted 50 bp downstream of human Vκ1-33 segment. The Vκ1-33/CBE replacement of Vκ3-2 was mediated by homologous recombination using a PGKneolox2DTA.2 (Addgene #13449) construct and two guide RNAs that target the mouse Vκ3-2 segment. The Cer/sis regulatory element in this ES cell line was deleted via the use of two guide RNAs that target sequences ~60 bp upstream and ~1 kb downstream of Cer/sis. A human TdT gene was expressed in this mouse model. The human TdT cDNA was cloned into CTV (Addgene #15912) construct in which the TdT expression was driven by CAG promotor and followed by a EGFP expression that mediated by an internal ribosome entry site (IRES) (*75*). The TdT expression cassette was inserted into the first intron of mouse Rosa26 gene which is on the same chromosome 6 with *Igκ* locus by homologous recombination. The sequences of guide RNAs used for targeting are listed in table S9. The modifications on the chromosome 12 (the *Igh* loci*)* and chromosome 6 (the *Igκ* and Rosa26 loci) were made independently in two ES clones. We performed blastocyst injection of these two ES clones, respectively, generating the $V_H1\text{-}2^{mVH\Delta/IGCR1\Delta}$-rearranging mouse model and the $V_H1\text{-}2^{IGCR1\Delta}$/$V\kappa1\text{-}33^{CS\Delta}$-rearranging mouse model. The $V_H1\text{-}2^{mVH\Delta/IGCR1\Delta}$/$V\kappa1\text{-}33^{CS\Delta}$-rearranging F1 mice were generated by cross-breeding of these two colonies. We then generated mice that were homozygous for each modification and used these mice for all experiments.

All mouse experiments were performed under protocol 20-08-4242R approved by the Institutional Animal Care and Use Committee of Boston Children's Hospital. Mice were maintained on a 14-h light, 10-h dark schedule in a temperature-controlled environment, with

food and water provided ad libitum. ES cells were grown on a monolayer of mitotically inactivated mouse embryonic fibroblasts (iMEF) in DMEM medium supplemented with 15% bovine serum, 20mM HEPES, 1x MEM nonessential amino acids, 2mM Glutamine, 100 units of Penicillin/Streptomycin, 100 mM ß-mercaptoethanol, 500 units/ml Leukemia Inhibitory Factor (LIF).

To characterize B cells in the homozygous $V_H1\text{-}2^{mVH\Delta/IGCR1\Delta}/V\kappa1\text{-}33^{CS\Delta}$ -rearranging mouse model, splenocytes were isolated from 5-8 week old mice and stained with the following antibodies: APC anti-B220 (eBioscience 17-0452-83); PE anti-Thy1.2 (eBioscience 12-0902-83); Bv605 anti-IgM (BioLegend 406523); Bv711 anti-IgD (BioLegend 405731).

Immunogens and Immunizations

HexaPro Spike immunogen was produced and purified as previously described (*76-79*). HexaPro Spike was stabilized by the introduction of six prolines in the S2 region and contained an HRV 3C-cleavable C-terminal twinStrepTagII-8×His tag to facilitate purification (PMID: 32703906). DNA encoding SARS-CoV-2 HexaPro Spike was synthesized (Genscript) and transiently transfected in FreeStyle 293F cells (Thermo Fisher) using 293Fectin (ThermoFisher). On day 5, cell culture supernatants were harvested by centrifugation of the culture followed by filtering through a 0.8-µm filter. Supernatant was concentrated down to 400 mL and Spike protein was purified by StrepTactin chromatography (IBA) using the manufacturer's buffers. Trimeric spike protein was further purified by Superose 6 size-exclusion chromatography (GE Healthcare) in 10 mM Tris pH 8, 500 mM NaCl. All purification steps were done at room temperature. Purified proteins were quantified and snap frozen in dry ice mixed with ethanol.

The VHH7-RBD immunogen was made as previously described (*80*). Briefly, VHH7-RBD DNA was synthesized (Integrated DNA Technologies) and assembled in the pVRC vector. Constructs were transfected into Expi293 cells (Thermo Fischer Scientific) using Polyethyleneimine "Max" (Polysciences). Cell cultures were maintained in Expi293 Media (Thermo Fischer Scientific) at 37°C for 4 day following transfection. Proteins were harvested by centrifugation at 5,000 Å~ g for 30 min at 4°C, followed by affinity chromatography with HisPur

Ni-NTA Resin (Thermo Fischer Scientific) and size exclusion chromatography with a Hi-Load 16/600 S75 column (Cytivia).

For each immunization, 200 ul of immunogen mix, containing 25 ug of filter-sterilized protein immunogen and 60 ug of Poly I:C in PBS, was injected into the peritoneum of each mouse. 8 weeks old mice were immunized twice with same immunogen, 4 weeks apart. Blood samples were collected one day before the first immunization and two weeks after each immunization. Spleens were collected 3 weeks after the second immunization.

Pseudovirus Neutralization Tests

The pseudovirus neutralization assay in 293T/ACE2 cells performed at Duke has been described in detail (*81*). and is a formally validated adaptation of the assay utilized by the Vaccine Research Center; the Duke assay is FDA approved for D614G. For measurements of neutralization, pseudovirus was incubated with 8 serial 5-fold dilutions of antibody samples (1:20 starting dilution using antibodies diluted to 1.0 mg/ml or 0.5 mg/ml) in duplicate in a total volume of 150 µl for 1 hr at 37°C in 96-well flat-bottom culture plates. 293T/ACE2-MF cells were detached from T75 culture flasks using TrypLE Select Enzyme solution, suspended in growth medium (100,000 cells/ml) and immediately added to all wells (10,000 cells in 100 µL of growth medium per well). One set of 8 wells received cells + virus (virus control) and another set of 8 wells received cells only (background control). After 71-73 hrs of incubation, medium was removed by gentle aspiration and 30 µl of Promega 1X lysis buffer was added to all wells. After a 10-minute incubation at room temperature, 100 µl of Bright-Glo luciferase reagent was added to all wells. After 1-2 minutes, 110 µl of the cell lysate was transferred to a black/white plate. Luminescence was measured using a GloMax Navigator luminometer (Promega). Neutralization titers are the inhibitory dilution (ID) of antibody at which RLUs were reduced by 50% (ID50) compared to virus control wells after subtraction of background RLUs.

The pseudovirus neutralization assays in 293T/ACE2/TMPRSS2 cells were performed as previously described (*82-84*). Briefly, human codon-optimized cDNA encoding SARS-CoV-2 spike glycoprotein of the WA-1/2020 and variants were synthesized by GenScript and cloned into eukaryotic cell expression vector pcDNA 3.1 between the *BamH*I and *Xho*I sites.

Pseudovirions were produced by co-transfection Lenti-X 293T cells with psPAX2(gag/pol), pTrip-luc lentiviral vector and pcDNA 3.1 SARS-CoV-2-spike-deltaC19, using Lipofectamine 3000. The supernatants were harvested at 48h post transfection and filtered through 0.45µm membranes and titrated using 293T/ACE2/TMPRSS2 cells (HEK 293T cells that express ACE2 and TMPRSS2 proteins). For the neutralization assay, 50 µL of SARS-CoV-2 S pseudovirions (counting ~200,000 relative light units) were pre-incubated with an equal volume of medium containing serial dilutions of mAbs at room temperature for 1h. Then 50 µL of virus-antibody mixtures were added to 293T-ACE2-TMPRSS2 cells (10^4 cells/50 µL) in a 96-well plate. The input virus with all SARS-CoV-2 strains used in the current study were the same (2 x 10^5 relative light units/50 µL/well). After a 3 h incubation, fresh medium was added to the wells. Cells were lysed 24 h later, and luciferase activity was measured using One-Glo luciferase assay system (Promega, Cat# E6130). The assay of each serum was performed in duplicate, and the 50% neutralization titer was calculated using Prism 9 (GraphPad Software). Controls included cells only, virus without any antibody and positive sera.

Antibody Affinity and Competition Assays by Surface Plasmon Resonance (SPR)

SPR screening and affinity measurements of monoclonal Fabs binding to SARS-CoV-2 spike proteins were performed using a Biacore S200 instrument (Cytiva, formerly GE Healthcare) in HBS-EP+ 1X running buffer and as described previously (*76*). The SARS-CoV-2 spike (S) proteins with a Strep tag were first captured onto a Streptavidin sensor chip to a level of 200-400 RU. For screenings, the Fabs were injected over the captured S proteins using the high performance injection mode at a flow rate of 30uL/min. Association phase was maintained at 180s injections of each Fab followed by a dissociation of 720s. After each dissociation phase, the S proteins and bound Fabs were removed from the sensor surface using a 30s regeneration pulse of Glycine pH1.5. For affinity measurement, the Fabs were injected over the captured S proteins using single cycle kinetics with the high performance injection mode at a flow rate of 50uL/min. Association phase was maintained at 120s injections of each Fab at increasing concentrations followed by a dissociation of 900s. After each dissociation phase, the CoV-S proteins and bound Fabs were removed from the sensor surface using a 30s regeneration pulse of Glycine pH1.5.

Results were analyzed using the Biacore S200 Evaluation software (Cytiva). A blank streptavidin surface along with blank buffer binding were used for double reference subtraction to account for non-specific protein binding and signal drift. Subsequent curve fitting analyses were performed using a 1:1 Langmuir model with a local Rmax for the Fabs or using the heterogeneous ligand model with local Rmax.

Antibody binding competition and blocking were measured by SPR following immobilization by amine coupling of monoclonal antibodies to CM5 and CM3 sensor chips (BIAcore/Cytiva) (*76*). Antibody blocking assays were performed by sequential high performance injections of Spike protein (20 µM) over mAb immobilized surfaces for 3 minutes at 30 µL/min immediately followed by a test Ab (200 µM) for 3 minutes at 30 µL/min. The dissociation of the antibody sandwich complex with the spike protein was monitored for 10 minutes with buffer flow and then a 24 second injection of Glycine pH2.0 for regeneration. Double reference subtraction was used to account for signal drift. Data analyses were performed with BIA-evaluation 4.1 software (BIAcore/Cytiva). The reported competition experiments are representative of two data sets.

NSEM Methods

To form the Fab-spike complex, Fab was mixed with spike at a 9:1 molar ratio and incubated at 37 °C for 1 h. The complex was then cross-linked for 5 min by diluting to 200 µg/ml spike concentration with 8 mM glutaraldehyde in HBS buffer (20 mM HEPES, 150 mM NaCl, 5 g/dL glycerol, pH 7.4), and then quenched for 5 min by addition of sufficient 1 M Tris stock to give 80 mM final Tris concentration. For negative stain, a portion of the sample was diluted in HBS buffer to 100 or 50 µg/ml and 5 µl applied to a glow-discharged carbon-coated EM grid for 10-12 second, then blotted, and stained with 2 g/dL uranyl formate for 1 min, and then blotted and air-dried. Grids were examined on a Philips EM420 electron microscope operating at 120 kV and nominal magnification of 49,000x, and ~100 images were collected on a 76 Mpix CCD camera at 2.4 Å/pixel. Images were analyzed by 2D class averages and 3D reconstructions calculated using standard protocols with Relion 3.0 (*85*).

Cryo-EM single particle analysis

Cryo-EM images were acquired on a Titan Krios electron microscope equipped with a Gatan K3 direct electron detector. We used cryo-SPARC (*86*) for particle picking, two-dimensional (2D) classification, three dimensional (3D) classification and refinement. To prepare cryo EM grids, the full-length G614 spike trimer (3.1 mg/ml)(*87*) and the SP1-77 Fab (7 mg/ml) were mixed at a molar ratio of 1:9 and incubated at room temperature for one hour. The complex was purified by gel filtration chromatography on a Superose 6 10/300 column (GE Healthcare, Chicago, IL) in a buffer containing 25 mM Tris-HCl, pH 7.5, 150 mM NaCl, 0.02% DDM. Both the mixed complex and purified complex were used for cryo grid preparation. 4.0 µl of the complex was applied to a 1.2/1.3 Quantifoil gold grid (Quantifoil Micro Tools GmbH), which had been glow discharged with a PELCO easiGlow™ Glow Discharge Cleaning system (Ted Pella, Inc.) for 60 s at 15 mA. Grids were immediately plunge-frozen in liquid ethane using a Vitrobot Mark IV (ThermoFisher Scientific), and excess protein was blotted away by using grade 595 filter paper (Ted Pella, Inc.) with a blotting time of 4 s, a blotting force of -12 at 4°C with 100% humidity. The grids were first screened for ice thickness and particle distribution. Selected grids were used to acquire images by a Titan Krios transmission electron microscope (ThermoFisher Scientific) operated at 300 keV and equipped with a BioQuantum GIF/K3 direct electron detector. Automated data collection was carried out using SerialEM version 3.8.6 (*88*) at a nominal magnification of 105,000× and the K3 detector in counting mode (calibrated pixel size, 0.83 Å) at an exposure rate of 13.761 electrons per pixel per second. Each movie add a total accumulated electron exposure of ~53.853 e-/Å^2, fractionated in 50 frames. Data sets were acquired using a defocus range of 0.5-2.2 µm.

Cryo-EM images were first processed in cryoSPARC v.3.3.1.(*86*) with the drift correction performed using the patch mode, and contrast transfer function (CTF) estimated by the patch mode as well. Motion corrected sums with dose-weighting were used for subsequent image processing. Templates for particle-picking were generated from a small number of manually picked particles and the template-based particle picking was then performed for all 28,899 recorded images with 7,657,522 particles extracted in total (box size 672Å, downsizing to 128Å). The particles were subjected to four rounds of 2D classification in cryoSPARC,

giving 3,894,421 good particles. A low-resolution negative-stain reconstruction of the Wuhan-Hu-1 (D614) sample was low-pass filtered to 40Å resolution and used as an initial model for 3D classification (*89*). The selected good particles were subjected to two rounds of heterogeneous 3D classification with six copies of the initial model as references in C1 symmetry. One major class (12.8%) with clear structural features was re-extracted to a smaller boxsize (480Å) and subjected to another two rounds of heterogeneous refinement with four copies of the initial model as references in C1 symmetry. There were two major classes, one representing the three-RBD-down S trimer bound with three SP1-77 Fabs and another representing the one-RBD-up S trimer in complex with three SP1-77 Fabs. The first class was subjected to one round of non-uniform refinement in C3 symmetry, giving a map at 2.9Å resolution from 250,319 particles. The second class was refined in C1 symmetry instead, giving a map at 3.1Å resolution from 209,249 particles. To further improve the overall resolution, both classes were subjected to local CTF refinement, resulting in a 2.7Å map for the complex with the S trimer in the three-RBD-down conformation, and a 2.9Å map for a 2.7Å map for the complex with the S trimer in the one-RBD-up conformation. To improve the density for the Fab and RBD, several different local refinements were performed with a soft mask covering a single RBD and the bound Fab. From the three-RBD-down complex, the local refinement gave a map at 3.2Å resolution; from the one-RBD-up complex, the local refinements gave a 3.3Å map for the Fab bound to the RBD in the down conformation, and a 4.8Å map for the Fab bound to the RBD in the up conformation. The best density maps were used for model building.

All resolutions were reported from the gold-standard Fourier shell correlation (FSC) using the 0.143 criterion. Density maps were corrected from the modulation transfer function of the K3 detector and sharpened by applying a temperature factor that was estimated using Sharpening Tools in cryoSPARC. Local resolution was also determined using cryoSPARC.

For model building, we used our G614 S trimer structures (PDB ID: 7KRQ and PDB ID: 7KRR (*90*)) as the initial templates for the spike protein, and the S309 antibody structure (PDB ID: 6WS6 (*91*)) for SP1-77 antibody. We predicted the SP1-77 Fab structure by AlphaFold (*92*), largely confirming our model building and the predicted structure also helped further improving some details of the final model. Several rounds of manual building were performed in Coot (*93*).

The models were refined in Phenix (*94*) against the 2.7Å (three-RBD-down) and 2.9Å (one-RBD-up) maps, respectively, as well as the 3.2Å map from the masked local refinement using the model containing a single RBD and the SP1-77 Fab. Iteratively, refinement was performed in both Phenix (real space refinement) and ISOLDE (*95*), and the Phenix refinement strategy included minimization_global, local_grid_search, and adp, with rotamer, Ramachandran, and reference-model restraints, using 7KRQ and 7KRR as the reference model. The refinement statistics are summarized in table S5. Structural biology applications used in this project were compiled and configured by SBGrid (*96*).

Generation of VSV-SARS-CoV-2 Atto 565

The generation of recombinant VSV chimeras expressing eGFP where the glycoprotein G was replaced with spike (S) protein Wuhan-Hu-1 strain (VSV-SARS-CoV-2) was previously described (*97*). VSV-SARS-CoV-2 chimera were obtained by infection of MA104 cells. Cells were grown in 15 to 20 150-mm dishes and infected at a multiplicity of infection (MOI) of 0.01. The supernatant containing the virus was collected 72 hours post infection and clarified by centrifugation at 1,000 x g for 10 min at 4ºC. A pellet with virus and extracellular particles was obtained by centrifugation in a Ti45 fixed-angle rotor at 72,000 x g (25,000 rpm) for 2 hours at 4ºC, then resuspended overnight in 0.5 mL PBS at 4ºC. This solution was layered on top of then pelleted through 15% sucrose-PBS solution by centrifugation in a SW55 swinging-bucket rotor at 148,000 x g (35,000 rpm) for 2 hours at 4ºC. The pellet was then resuspended overnight in 0.4 mL PBS at 4ºC, layered on top of a 15 to 45% sucrose-PBS linear gradient and subjected to centrifugation in a SW55 swinging-bucket rotor at 194,000 x g (40,000 rpm) for 1.5 hours at 4ºC. The predominant light scattering band located in the lower one-third of the gradient and containing the virions was removed by side puncture of the gradient tube. Approximately 0.3 ml of this solution was mixed with 25 ml of PBS and subjected to centrifugation in a Ti60 fixed-angle rotor at 161,000 x g (40,000 rpm) for 2 hours at 4ºC. The final pellet was resuspended overnight in 0.2 - 0.5 mL PBS aliquots and stored at -80°C.

Stock concentration of VSV-SARS-CoV-2 at a concentration of ~150 µg/mL viral RNA was conjugated with Atto565-NHS ester (Sigma-Aldrich, cat.72464) as previously described

(*98*). Virus stocks in 200 µL were adjusted to 0.1 M NaHCO3 (pH 8.3). Atto565 NHS esters were resuspended in anhydrous DMSO (Sigma) and then aliquoted to and dried down into 0.25 µg/mL stocks under house vacuum and then stored at -20ºC. Virus stock was added to dried Atto565 NHS ester and incubated at room temperature for 1 hour in the dark. After 1 hour, Tris pH 8.0 to a final concentration of 200 mM was added to quench the reaction. Free label was then separated from labeled virus using a 0.5 mL Piece Zeba spin desalting column with a 40 kDa molecular weight cut off. Labeled virus was stored at 4ºC and used within 1 week of labeling.

Infection assays were done with cells plated one day before the experiment and used for the infection assays with a final density of ~ 80% confluency as previously described (*97*). VSV -SARS-CoV-2-Atto 565 was incubated with the indicated concentration of antibody for 1 hour at 37°C. Virus was then added to the cells with indicated concentration of antibody for 1 hour, followed by 3 washes with cell culture media (DMEM, 10% FBS, 25 mM HEPES, pH 7.4), and then incubation for 7 hours at 37°C and 10% CO_2 in DMEM containing 25 mM HEPES, pH 7.4. At the end of the 7-hr incubation, cells were treated for 30 sec with 5 µg/mL WGA-Alexa647 in PBS to define the cell outline, then fixed with 4% PFA in PBS for 15 minutes at room temperature and imaged within 24 hours using a spinning-disk confocal microscope equipped with a 40x oil NA=1.4 objective (1 pixel = 0.33 µm) to acquire random fields, each containing a Z-stack of 20 consecutive optical planes taken 1 µm apart. Cells were scored as infected if the cytosolic eGFP fluorescence was 1.4 times that of the background of uninfected cells from the control experiment without virus.

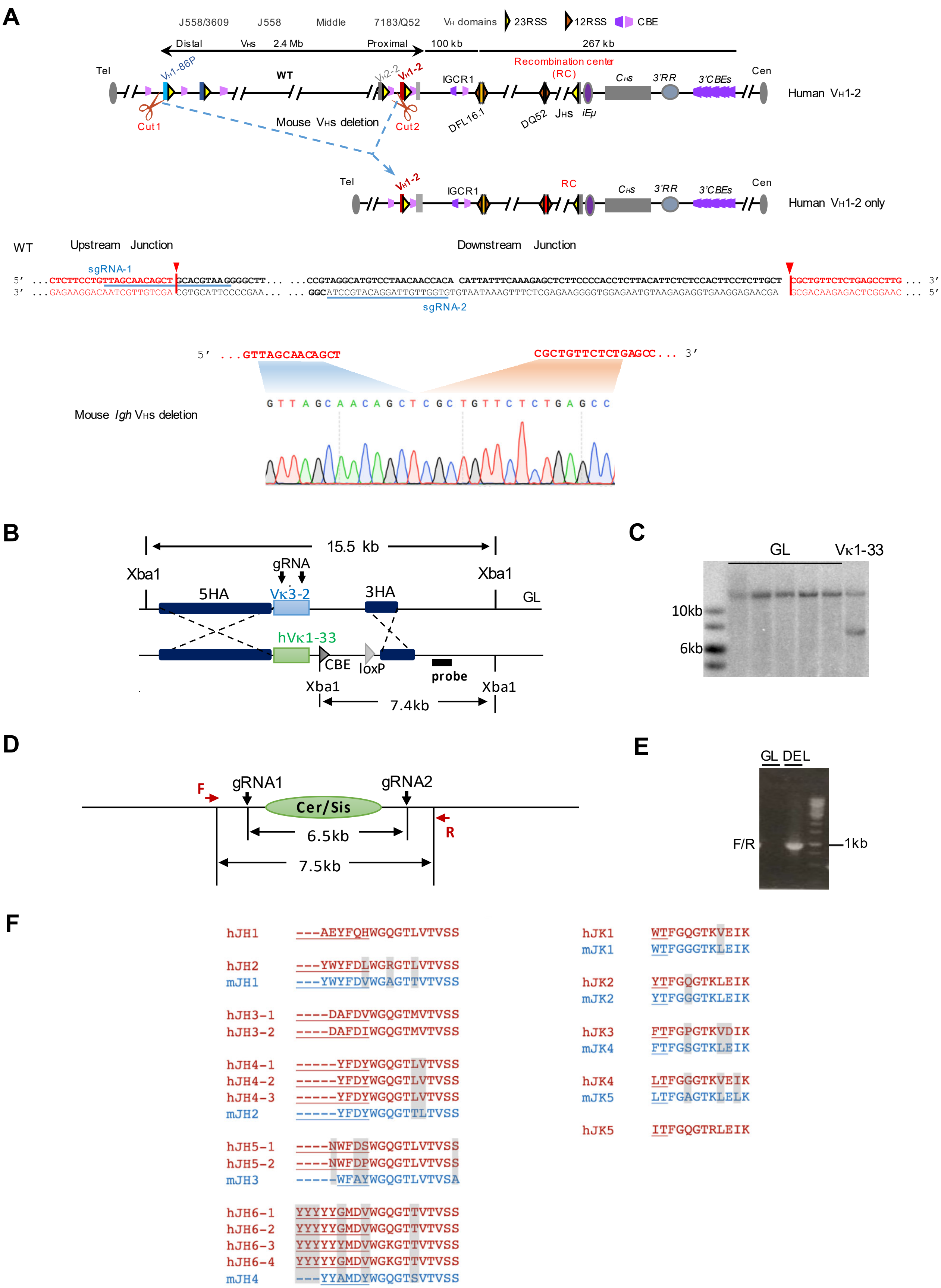
A
J558/3609
J558
Middle
7183/Q52
VH domains
23RSS
12RSS
CBE
Distal
VHS
2.4 Mb
Proximal
100 kb
267 kb
Recombination center
(RC)
Tel
VH1-86P
WT
VH2-2
VH1-2
IGCR1
CHS
3'RR
3'CBEs
Cen
Human VH1-2
Cut1
Mouse VHS deletion
Cut2
DFL16.1
DQ52
JHS
iEμ
Tel
VH1-2
IGCR1
RC
CHS
3'RR
3'CBEs
Cen
Human VH1-2 only
WT
Upstream Junction
Downstream Junction
sgRNA-1
5' ...CTCTTCCTGTTAGCAACAGCT GCACGTAAGGGGCTT... ...CCGTAGGCATGTCCTAACAACCACA CATTATTTCAAAGAGCTCTTCCCCACCTCTTACATTCTCTCCACTTCCTCTTGCT CGCTGTTCTCTGAGCCTTG... 3'
3' ...GAGAAGGACAATCGTTGTCGA CGTGCATTCCCCGAA... ...GGCATCCGTACAGGATTGTTGGTGTGTAATAAAGTTTCTCGAGAAGGGGTGGAGAATGTAAGAGAGGTGAAGGAGAACGA GCGACAAGAGACTCGGAAC... 5'
sgRNA-2
5' ...GTTAGCAACAGCT
CGCTGTTCTCTGAGCC... 3'
Mouse Igh VHS deletion
G T T A G C A A C A G C T C G C T G T T C T C T G A G C C
B
15.5 kb
Xba1
gRNA
Xba1
5HA
Vκ3-2
3HA
GL
hVκ1-33
CBE
loxP
probe
Xba1
Xba1
7.4kb
C
GL
Vκ1-33
10kb
6kb
D
F
gRNA1
gRNA2
Cer/Sis
R
6.5kb
7.5kb
E
GL DEL
F/R
1kb
F
hJH1 ---AEYFQHWGQGTLVTVSS
hJH2 ---YWYFDLWGRGTLVTVSS
mJH1 ---YWYFDVWGAGTTVTVSS
hJH3-1 ----DAFDVWGQGTMVTVSS
hJH3-2 ----DAFDIWGQGTMVTVSS
hJH4-1 -----YFDYWGQGTLVTVSS
hJH4-2 -----YFDYWGQGTLVTVSS
hJH4-3 -----YFDYWGQGTLVTVSS
mJH2 -----YFDYWGQGTTLTVSS
hJH5-1 ----NWFDSWGQGTLVTVSS
hJH5-2 ----NWFDPWGQGTLVTVSS
mJH3 -----WFAYWGQGTLVTVSA
hJH6-1 YYYYYGMDVWGQGTTVTVSS
hJH6-2 YYYYYGMDVWGQGTTVTVSS
hJH6-3 YYYYYYMDVWGKGTTVTVSS
hJH6-4 YYYYYGMDVWGKGTTVTVSS
mJH4 ---YYAMDYWGQGTSVTVSS
hJK1 WTFGQGTKVEIK
mJK1 WTFGGGTKLEIK
hJK2 YTFGQGTKLEIK
mJK2 YTFGGGTKLEIK
hJK3 FTFGPGTKVDIK
mJK4 FTFGSGTKLEIK
hJK4 LTFGGGTKVEIK
mJK5 LTFGAGTKLELK
hJK5 ITFGQGTRLEIK

Figure S1. Generation of the V_H1-2/Vκ1-33-rearranging mouse model. (**A**) The diagram illustrated the deletion of 2MB upstream region of human V_H1-2 that contain all mouse V_Hs. The deletion was mediated by two gRNAs (green). The deleted allele was screened by PCR. The sequencing result was shown in the bottom. (**B**) The diagram, not drawn to scale, illustrated the restriction digests and Southern probe that were used to differentiate the region before (GL) and after Vκ1-33 replacement. (**C**) Southern analysis of positive Vκ1-33 ES clones that showed in (**B**). (**D**) The diagram illustrates the deleted region of Cer/sis regulatory element targeted by two gRNAs and PCR primers that were used to identify the deleted clones. (**E**) PCR analysis of ES clones with Cer/sis deletion. (**F**) Similarities of amino acid sequences between mouse Js and human Js. Human Js were shown in red, and mouse Js were shown in blue. The alignments between human Js and mouse Js were mainly based on the sequences underlined at 5' end that contribute to the CDR3 sequence. The different amino acids between mouse Js and human Js were filled in grey.

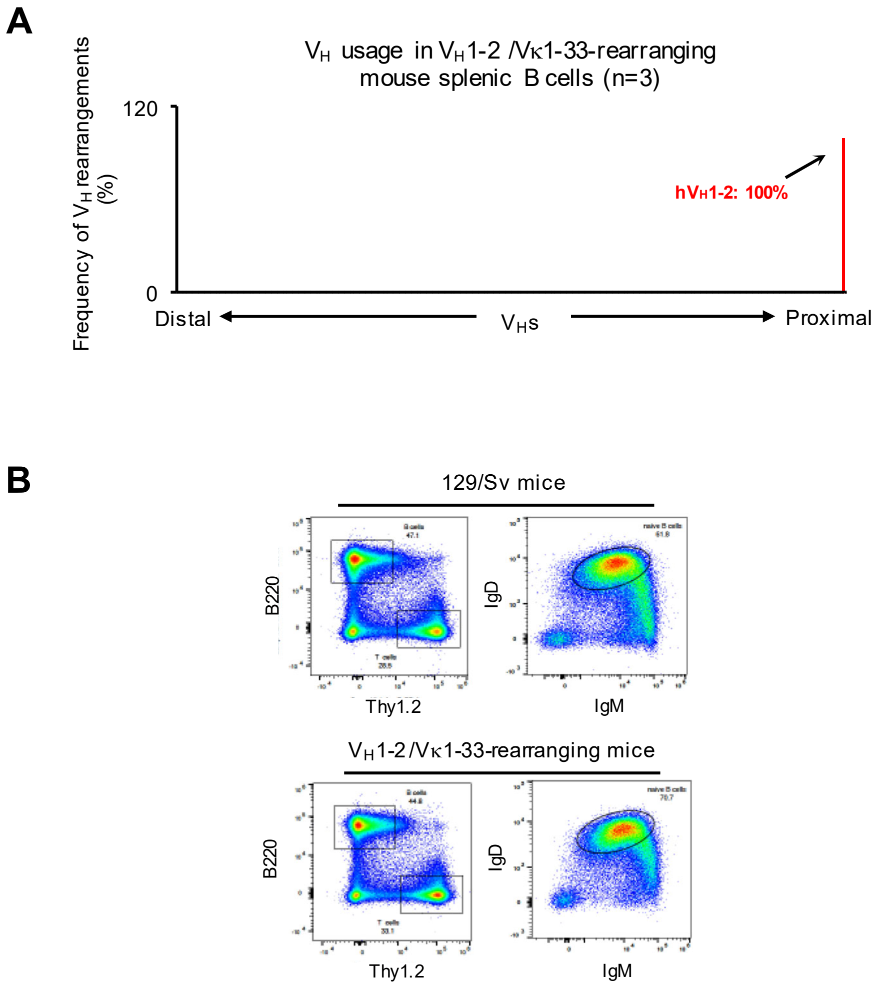

Figure S2. Characterization of the V_H1-2/Vκ1-33-rearranging mouse. (**A**) HTGTS-rep-seq analysis of V_H usage in $V_H1\text{-}2^{mVH\Delta/IGCR1\Delta}$-rearranging mouse splenic B cells. The x axis listed all functional V_Hs from the distal to the *D*-proximal end. The histogram displayed the percent usage of each V_H among all productive V_HDJ_H rearrangements. Data were mean ± SD of 3 different mice. The number of reads for each V_H was shown in Table S1. (**B**) FACS analyses of splenic B cell and T cell populations from wild-type 129/Sv and V_H1-2/Vκ1-33-rearranging mice. They were representative of three biological replicates.

A

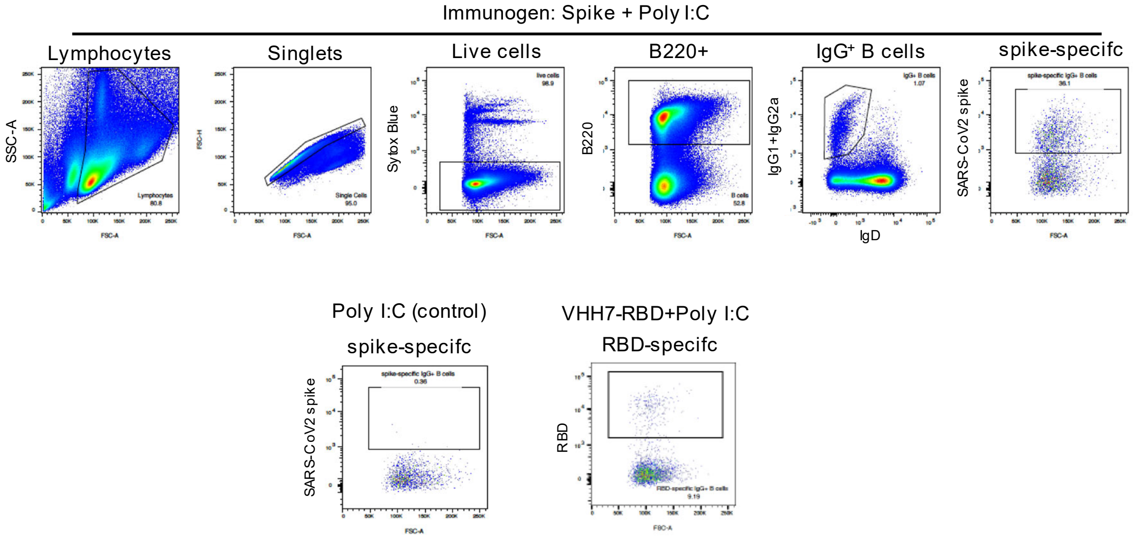

B

Binding Assay (EC_{50}: ng/ml)

	SARS_ CoV-2 Spike	SARS_ CoV-2 RBD	SARS_ CoV-2 NTD
SP1-77	9	14	>10,000
SP2-125	16	>10,000	>10,000
SP3-46	29	>10,000	>10,000
SP4-87	20	>10,000	>10,000
VHH7-3-66	4	3	>10,000
VHH7-5-82	2	3	>10,000
VHH7-7-53	3	3	>10,000
VHH7-8-59	6	4	>10,000
VHH7-10-19	4	5	>10,000

Binding affinity
EC_{50} (ng/ml)
<10
10-100
100-1,000
1,000-10,000
>10,000

Figure S3. Immunizing the mouse model with SARS-CoV-2 spike or RBD elicited V_H1-2/Vκ1-33 antibodies. (**A**) Gating strategy for single cell sorting of SARS-CoV2 spike-specific or RBD-specific IgG^+ B cells after immunization. The upper panel showed the mice immunized with SARS-CoV2 spike plus poly I:C adjuvant. The bottom panels showed the mice immunized with poly I:C (control) or VHH7-RBD plus poly I:C adjuvant. (**B**) Table showed the binding affinities of nine mAbs to the SARS-CoV-2 spike protein, RBD, NTD. Data were representative of two independent experiments. EC_{50} values were color-coded based on the key shown at the right.

A

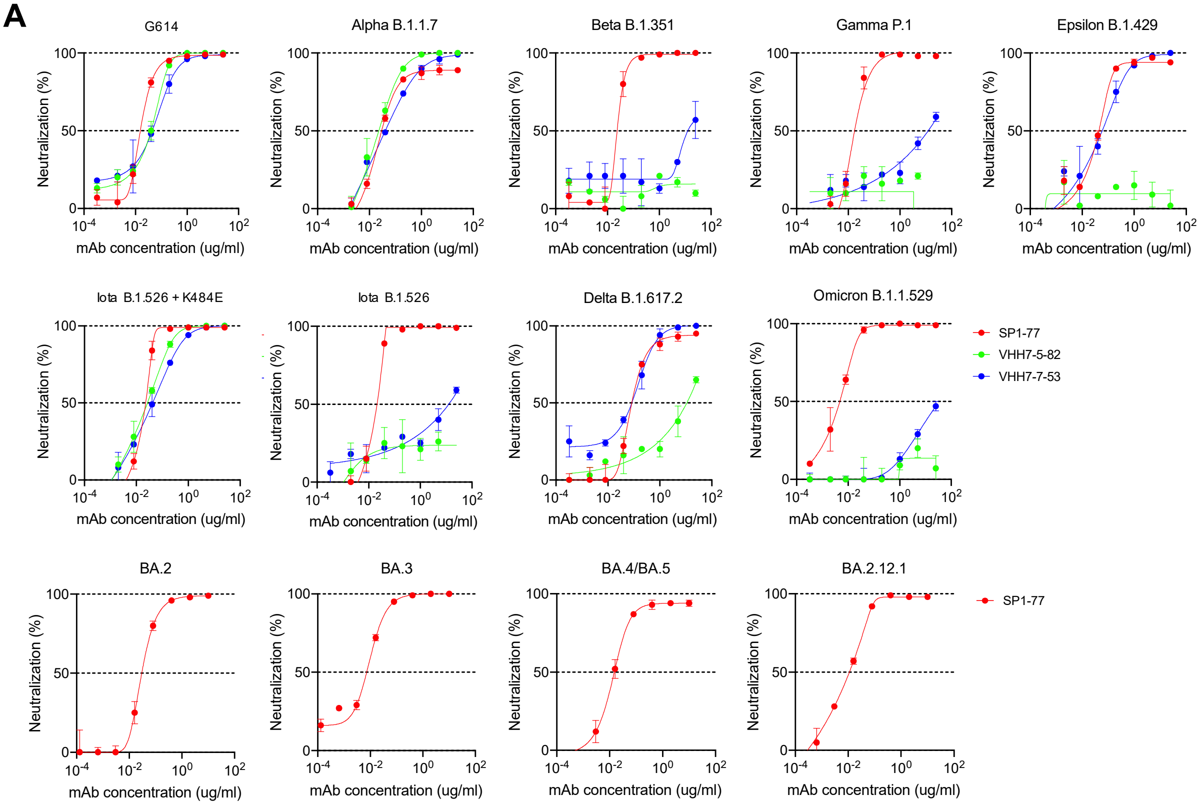

B

Pseudotyped Virus Neutralization of Monoclonal Antibodies – 293T/ACE2/TMPRSS2 (IC_{50}: ng/ml)

	Prototype	Alpha	Beta	Gamma	Epsilon	Iota	Delta	Omicron
	WA-1	B.1.1.7	B.1.351	P.1	B.1.429	B.1.526	B.1.617.2	BA.1
SP1-77	47	77	13	70	66	16	556	39
VHH7-5-82	82	81	>10,000	>10,000	>10,000	>10,000	>10,000	>10,000
VHH7-7-53	162	127	>10,000	>10,000	126	>10,000	131	>10,000

Neutralization titer IC_{50} (ng/ml)
- <10
- 10-100
- 100-1,000
- 1,000-10,000
- >10,000

C

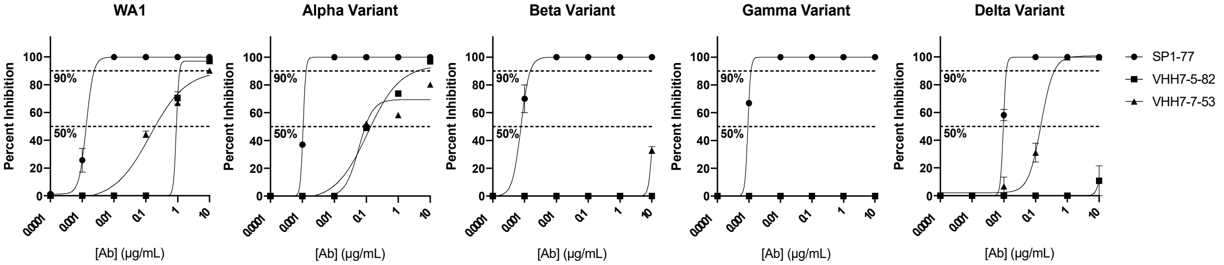

D

Pseudotyped Virus Neutralization of Monoclonal Antibodies – 293T/ACE2 (IC_{50}: ng/ml)

	Prototype		Delta		Omicron			
	G614		B.1.617.2		B.1.1.529 (BA.1)		BA.2	
	IC_{50}	IC_{80}	IC_{50}	IC_{80}	IC_{50}	IC_{80}	IC_{50}	IC_{80}
SP1-77 (human J)	26	86	91	207	4	19	19	80
SP1-13 (human J)	28	113	35	249	7	67	40	188
SP1-62 (human J)	28	125	47	282	2	47	48	316
SP1-73 (human J)	44	147	84	350	8	46	41	237
SP1-96 (human J)	22	76	66	166	14	45	30	138

Neutralization titer IC_{50} (ng/ml)
- <10
- 10-100
- 100-1,000
- 1,000-10,000
- >10,000

Figure S4. Neutralization data of three mAbs against SARS-CoV-2 variants. (**A**) Neutralization Curves of SARS-CoV-2 pseudoviruses displaying S proteins by three V_H1-2/Vκ1-33 mAbs. Data were done in 293T/ACE2 cells and also shown in **Fig. 3A**. Data were representative of one independent experiments with two technical replicates. (**B**) Table showed the neutralization activities of three mAbs against VOCs and VOIs in pseudovirus neutralization assays. These assays were done independently in 293T/ACE2/TMPRSS2 cells. Compared to the neutralization data done in 293T/ACE2 cells, IC_{50} values were at the similar levels for all VOCs and VOIs except Delta which was neutralized less potently. Data were representative of one independent experiment with two technical replicates. IC_{50} values were color-coded based on the key shown at the right. (**C**) Neutralization Curves of SARS-CoV-2 virus by three V_H1-2/Vκ1-33 mAbs in PRNT experiment. Data were done in 293T/ACE2 cells and also shown in Fig. 3B. Data were representative of one independent experiments with two technical replicates. (**D**) Table showed the neutralization activities of humanized SP1-77 and the other 4 antibodies in SP1 clonal lineage against VOCs in pseudovirus neutralization assays. These assays were done in 293T/ACE2 cells. Data were representative of two independent experiments. Each independent experiment contained 2 technical replicates. IC_{50} values were color-coded based on the key shown at the right.

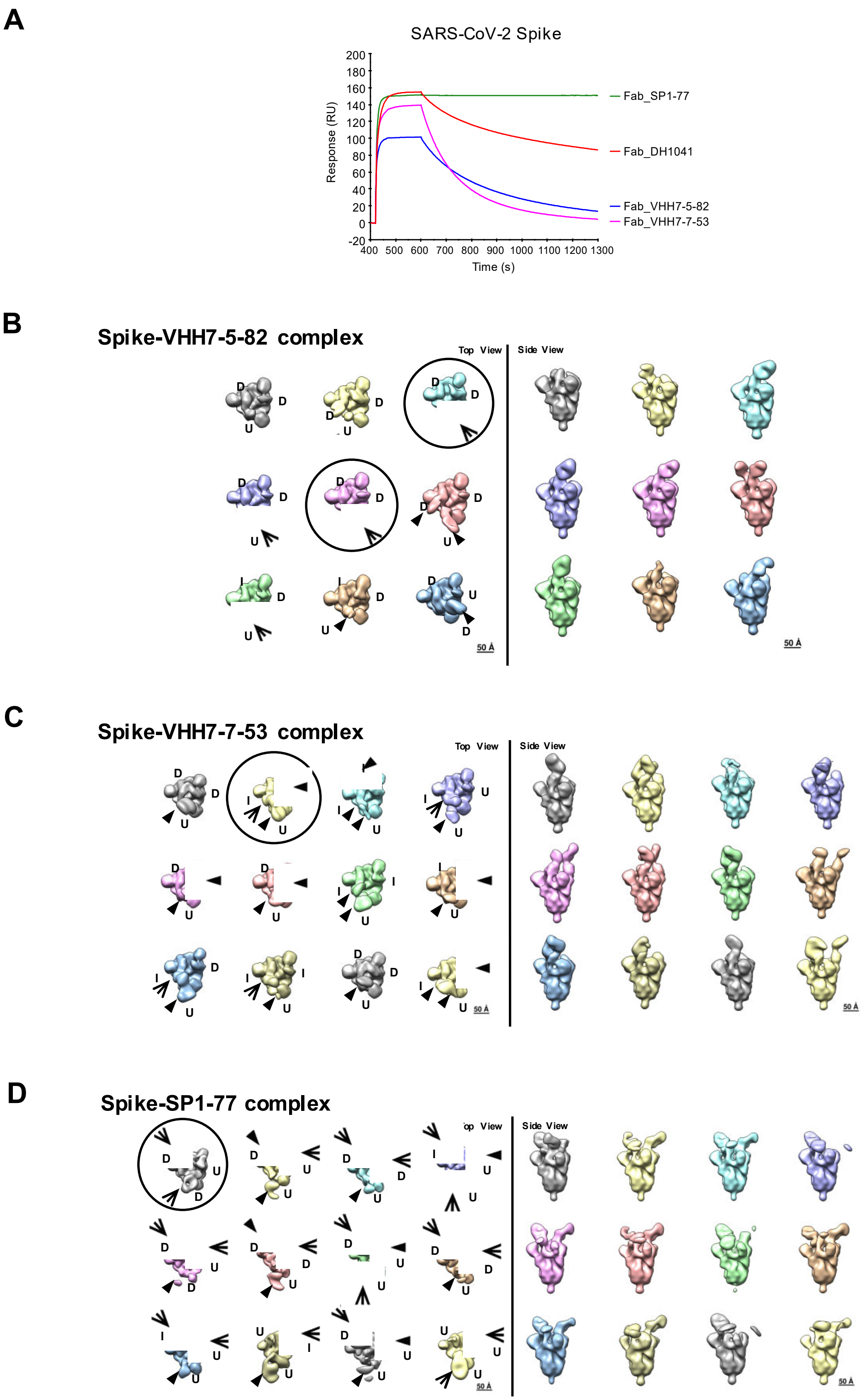
A
SARS-CoV-2 Spike
Response (RU)
Time (s)
Fab_SP1-77
Fab_DH1041
Fab_VHH7-5-82
Fab_VHH7-7-53
B
Spike-VHH7-5-82 complex
Top View
Side View
50 Å
C
Spike-VHH7-7-53 complex
Top View
Side View
50 Å
D
Spike-SP1-77 complex
Side View
50 Å

Figure S5. Negative stain electron microscopy analysis of S complexes with three V_H1-2/Vκ1-33 neutralizing antibodies. (**A**) SPR data measuring the binding affinity between monoclonal Fabs and SARS-CoV-2 spike proteins. Each coronavirus spike (S) protein was captured on Streptavidin sensor chip and Fabs were screened at 50nM. A blank streptavidin surface along with blank buffer binding were used for double reference subtraction to account for non-specific protein binding and signal drift. Data was representative of two independent experiments. (**B**) A 3D classification of the spike-VHH7-5-82 complex data, with 158,101 particles sorted into 9 classes, shown in top and side views. (**C**) A 3D classification of the spike- VHH7-7-53 complex data, with 187,262 particles sorted into 12 classes, shown in top and side views. (**D**) A 3D classification of the spike-SP1-77 complex data, with 230,136 particles sorted into 12 classes, shown in top and side views. In the top view of B-D, arrows indicate Fabs, and arrowheads indicate weak or partial Fab density; RBDs were visually assessed and assigned as up (U), down (D) or intermediate (I); and the circles indicated the selected (or combined) classes used for the final 3D reconstructions shown in **Fig. 3C**.

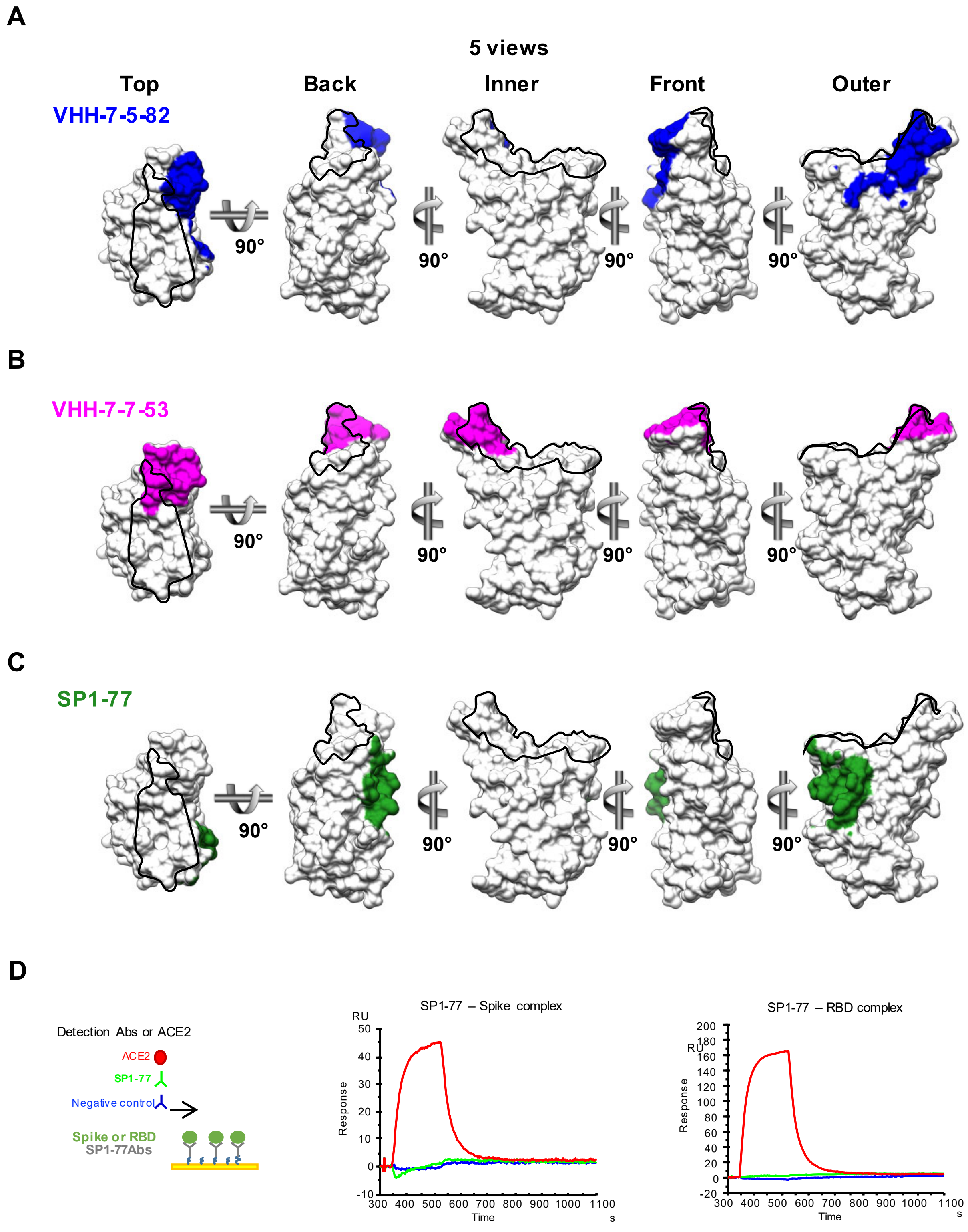
A
5 views
Top
Back
Inner
Front
Outer
VHH-7-5-82
90°
B
VHH-7-7-53
C
SP1-77
D
Detection Abs or ACE2
ACE2
SP1-77
Negative control
Spike or RBD
SP1-77Abs
SP1-77 – Spike complex
SP1-77 – RBD complex
RU
Response
Time
s

Figure S6. Three V_H1-2/Vκ1-33 neutralizing antibodies bound to distinct regions of RBD. (**A-C**) Binding footprint of Fabs of three V_H1-2/Vκ1-33 neutralizing antibodies, VHH7-5-82 (**A**), VHH7-7-53 (**B**), and SP1-77 (**C**) shading on the RBD in surface representation. The RBD surface was represented in 5 views (top, back, inner, front and outer). Black outline indicated the ACE2 RBM. RBD was shown as gray. (**D**) SP1-77 can not block ACE2 binding to the spike protein and RBD. The left panel showed the diagram of SPR assay. The middle panel showed the binding of ACE2, SP1-77 antibody and negative control (red, green and blue, respectively) to preformed SP1-77 - spike complex. The right panel showed the binding of ACE2, SP1-77 antibody and negative control (red, green and blue, respectively) to preformed SP1-77 - RBD complex.

A

Name	Heavy V Gene	Light V Gene	CDRH3	CDRL3
**SP1-77**	IGHV1-2	IGKV1-33	ARDRVLYGRSFGWYFDV	QQYDNLPT
**VHH7-5-82**	IGHV1-2	IGKV1-33	ARDRVGQRFPY	QQYDNLWT
**VHH7-7-53**	IGHV1-2	IGKV1-33	ARRNCDY	QQYDNLPYT
**Fab2-43**	IGHV1-2	IGLV2-14	ARGLGVGCSGGNCYLDYYYMDV	SSYTSSSTWV
**Fab2-15**	IGHV1-2	IGLV2-14	ARGGSRCSGGNCYGWAYDAFDI	SSYTSSSTFV
**S2-M11**	IGHV1-2	IGKV3-20	ARAAPFYDFWSGYSYFDY	QQYGSSAWT
**H4**	IGHV1-2	IGKV2-40	ARVPYCSSTSCHRDWYFDL	MQRIEFPLT
**COVOX-316**	IGHV1-2	IGLV2-8	ARDMAFSMVRGSFDY	SSYAGSNHWV
**C121**	IGHV1-2	IGLV2-23	ARAPLFPTGVLAGDYYYYGMDV	CSYAGSSTLV
**Fab2-4**	IGHV1-2	IGLV2-8	ARDRSWAVVYYYMDV	SSYAGSNNLV
**CV05-163**	IGHV1-2	IGKV3-11	AREVMVRGALPPYGMDV	QQRSNWPPVT
**REGN10989**	IGHV1-2	IGLV2-14	ARGSRYDWNQNNWFDP	SSFTTSSTVV

B

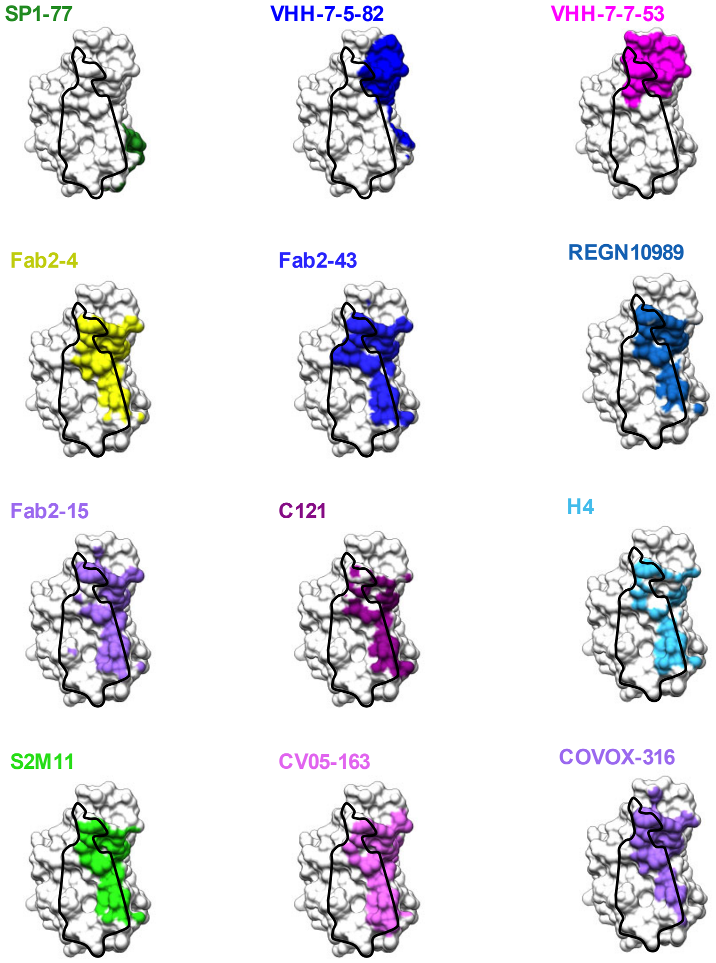

Figure S7. Epitope analysis and comparison of the previously characterized V_H1-2-based monoclonal antibodies. (**A**) Table showed a list of V_H1-2-based mAbs for which high-resolution structures have been published with various HC CDR3s and LCs. The three antibodies identified in our mouse model were labeled in red. (**B**) Comparisons of footprints on RBD between our three antibodies and nine previously characterized V_H1-2-based monoclonal antibodies. Those nine previously characterized V_H1-2-based antibodies show a similar RBD binding epitope that is distinct from where our three antibodies bound.

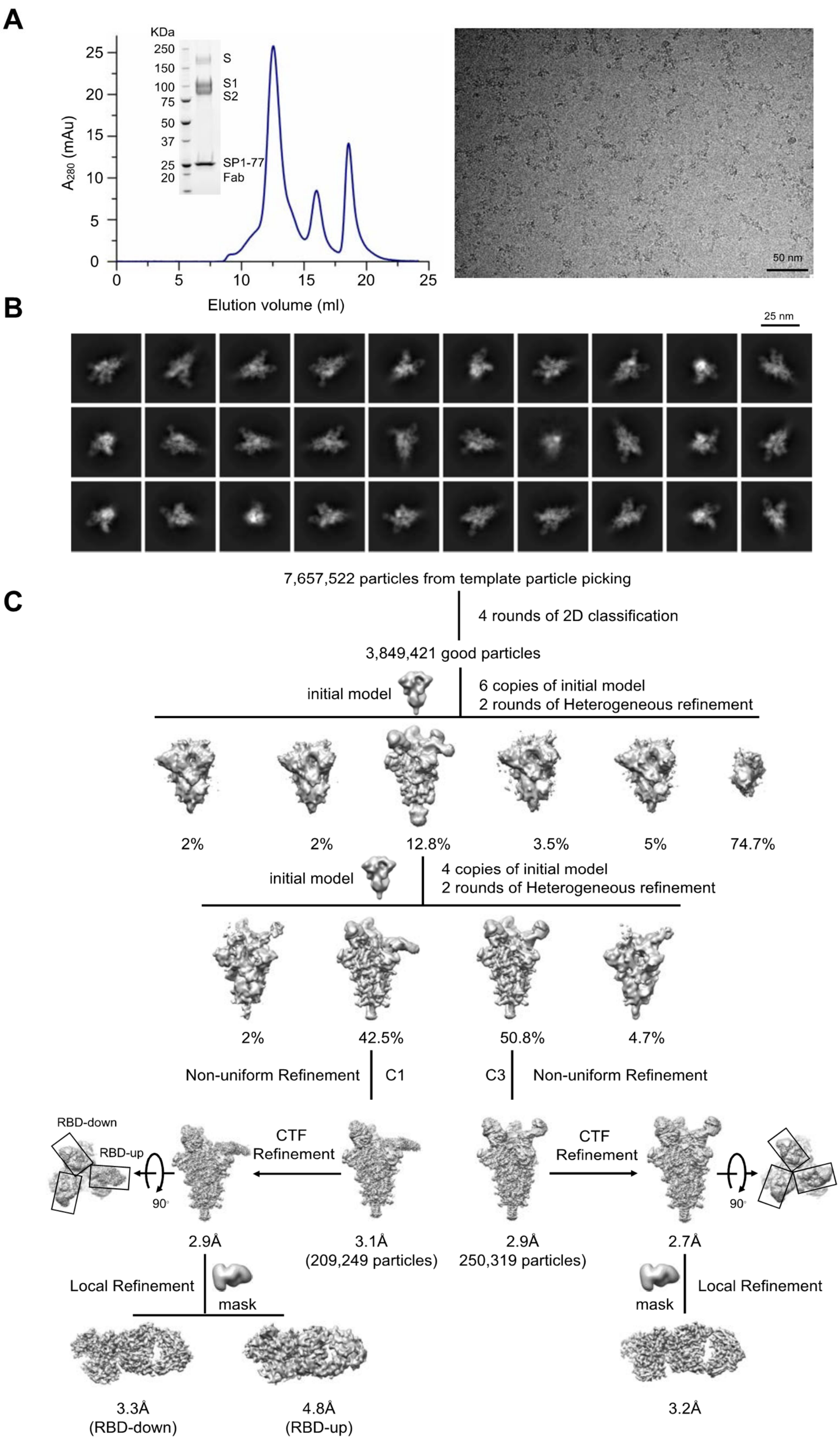

A
KDa
250
150
100
75
50
37
25
20
S
S1
S2
SP1-77
Fab
A280 (mAu)
Elution volume (ml)
50 nm
B
25 nm
C
7,657,522 particles from template particle picking
4 rounds of 2D classification
3,849,421 good particles
initial model
6 copies of initial model
2 rounds of Heterogeneous refinement
2%
2%
12.8%
3.5%
5%
74.7%
initial model
4 copies of initial model
2 rounds of Heterogeneous refinement
2%
42.5%
50.8%
4.7%
Non-uniform Refinement
C1
C3
Non-uniform Refinement
RBD-down
RBD-up
CTF Refinement
CTF Refinement
90°
90°
2.9Å
3.1Å
(209,249 particles)
2.9Å
250,319 particles)
2.7Å
Local Refinement
mask
mask
Local Refinement
3.3Å
(RBD-down)
4.8Å
(RBD-up)
3.2Å

Figure S8. Purification of the full-length G614 S trimer/SP1-77 Fab complex and cryo-EM analysis. (**A**) Left, the complex of the full-length G614 S trimer and SP1-77 Fab was resolved by gel-filtration chromatography on a Superose 6 column. First peak, the S trimer-Fab complex used for the cryo-EM study; second peak, the dissociated postfusion S2 trimer mixed with the monomeric S1 in complex with Fab; and third peak, free Fab. Inset, peak fractions were analyzed by Coomassie stained SDS-PAGE. Right, representative motion-corrected micrograph of the vitrified complex purified by gel-filtration chromatography. (**B**) 2D class averages (box dimension: 396Å) of the cryo-EM images of the G614 S trimer/SP1-77 Fab complex from cryoSPARC. (**C**) Data processing workflow for structure determination.

C3 symmetry, RBD-down G614 with SP1-77 Fab
250,319 particles
GSFSC Resolution 2.68Å

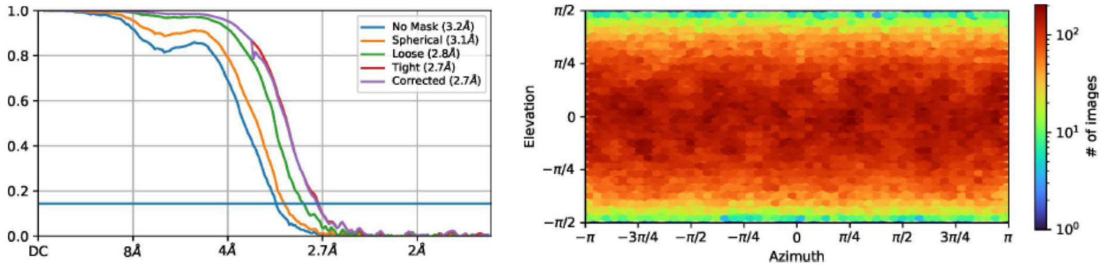

C1 symmetry, one RBD-up G614 with SP1-77 Fab
209,249 particles
GSFSC Resolution 2.92Å

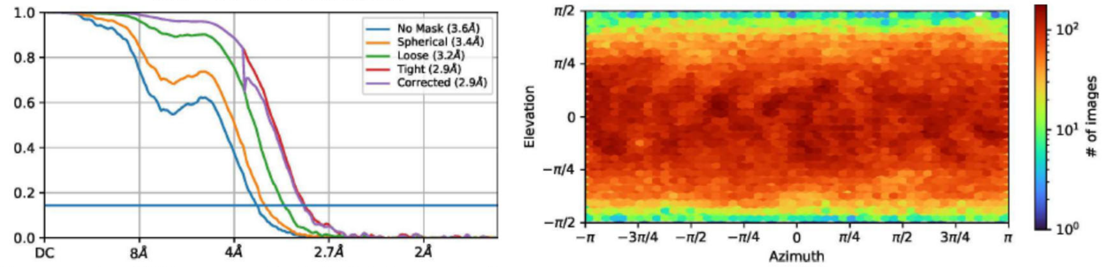

C1 symmetry, RBD-down with SP1-77 Fab local refine
250,319 particles
GSFSC Resolution 3.23Å

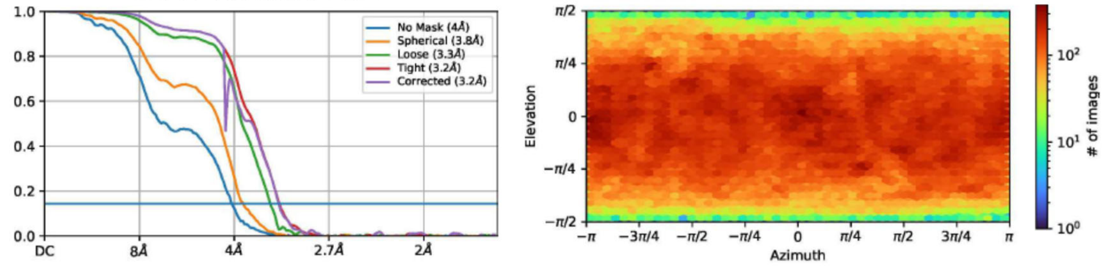

C1 symmetry, RBD-down with SP1-77 Fab local refine
209,249 particles
GSFSC Resolution 3.31Å

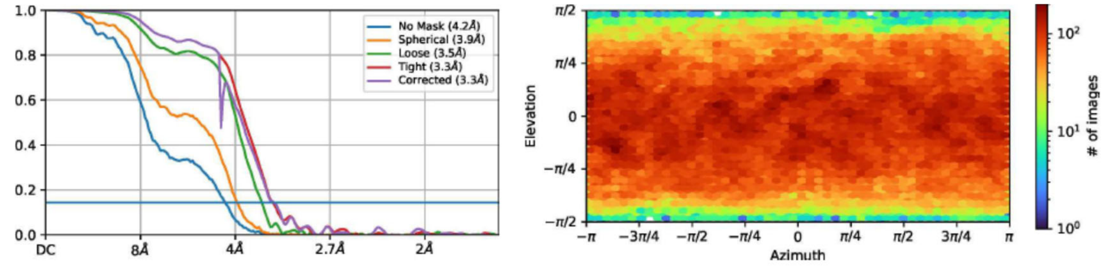

C1 symmetry, RBD-up with SP1-77 Fab local refine
209,249 particles
GSFSC Resolution 4.84Å

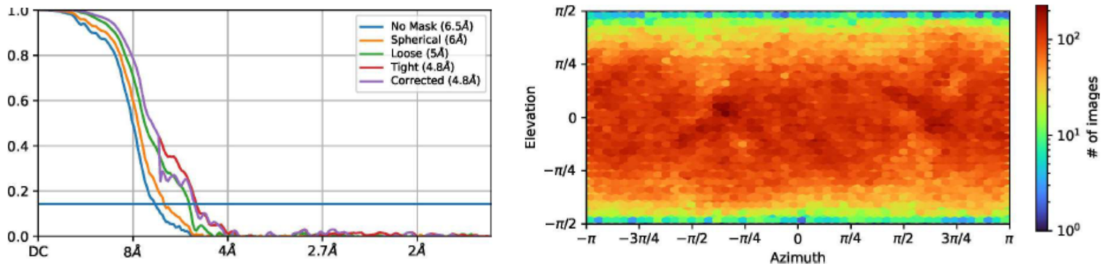

Figure S9. Cryo-EM structure validation of the G614 S trimer/SP1-77 Fab complex. FSC curves and the viewing direction distribution plot for the S trimer-Fab complex. From top to bottom: the complex of the G614 S trimer in the three-RBD-down conformation and three SP1-77 Fabs; the complex of the G614 S trimer in the one-RBD-up conformation and three SP1-77 Fabs; masked local refinement of the Fab in complex with the RBD from the G614 S trimer in the three-RBD-down conformation; masked local refinement of the Fab in complex with the RBD in the up conformation from the G614 S trimer in the one-RBD-up state; masked local refinement of the Fab in complex with the RBD in the down conformation from the G614 S trimer in the one-RBD-up state.

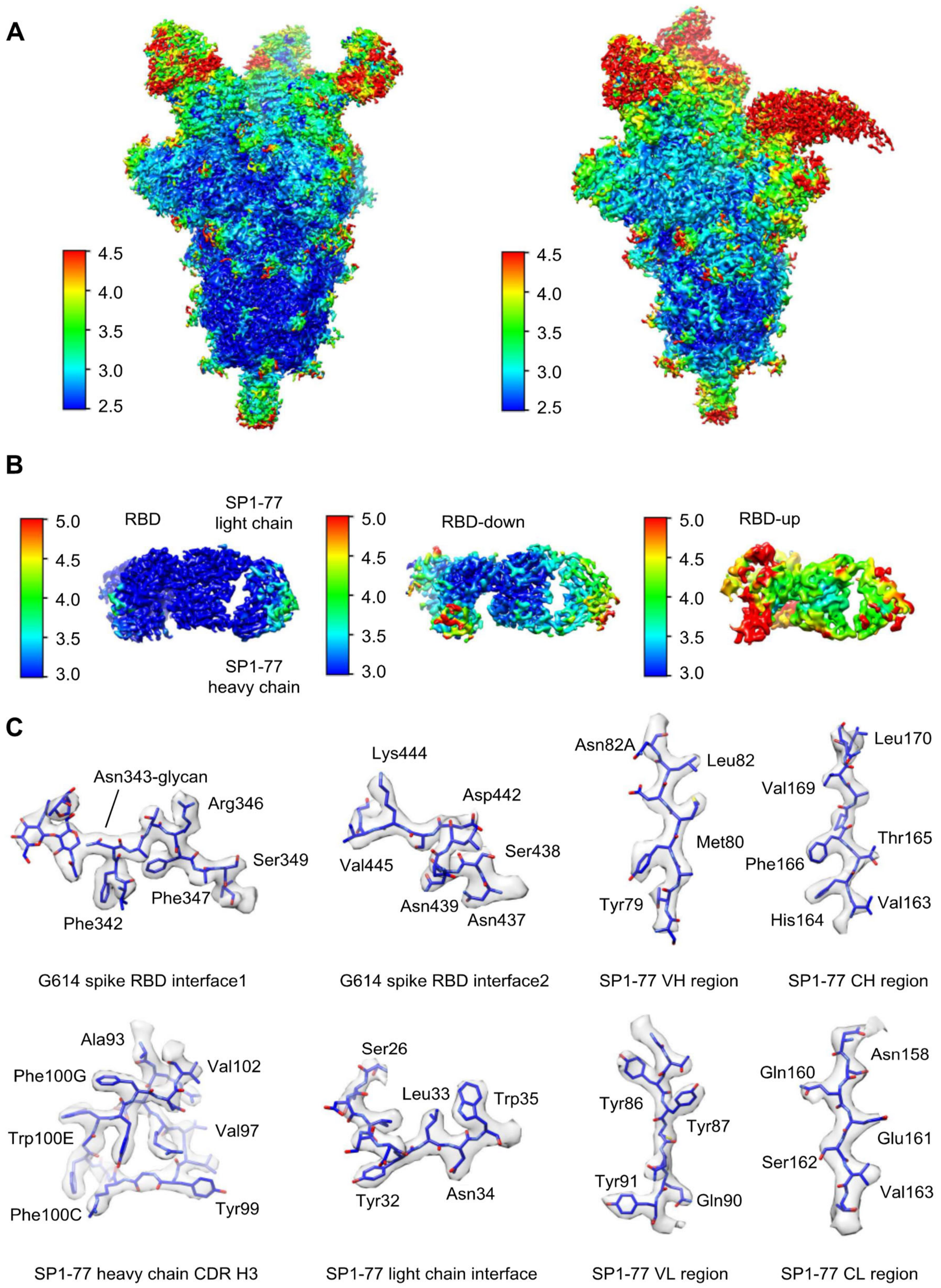
A
4.5
4.0
3.5
3.0
2.5
4.5
4.0
3.5
3.0
2.5
B
5.0
4.5
4.0
3.5
3.0
RBD
SP1-77
light chain
SP1-77
heavy chain
5.0
4.5
4.0
3.5
3.0
RBD-down
5.0
4.5
4.0
3.5
3.0
RBD-up
C
Asn343-glycan
Arg346
Ser349
Phe347
Phe342
Lys444
Asp442
Ser438
Val445
Asn439
Asn437
Asn82A
Leu82
Met80
Tyr79
Leu170
Val169
Thr165
Phe166
Val163
His164
G614 spike RBD interface1
G614 spike RBD interface2
SP1-77 VH region
SP1-77 CH region
Ala93
Val102
Phe100G
Val97
Trp100E
Phe100C
Tyr99
Ser26
Leu33
Trp35
Tyr32
Asn34
Tyr86
Tyr87
Tyr91
Gln90
Asn158
Gln160
Glu161
Ser162
Val163
SP1-77 heavy chain CDR H3
SP1-77 light chain interface
SP1-77 VL region
SP1-77 CL region

Figure S10. Analysis of the the G614 S trimer/SP1-77 Fab complex structure. (**A**) 3D reconstructions of SP1-77 Fab in complex with the G614 S trimer in the three-RBD-down and one-RBD-up conformations, respectively, were colored according to local resolution estimated by cryoSPRAC. (**B**) 3D reconstructions from masked local refinement of the Fab in complex with a single RBD from the G614 S trimer in the three-RBD-down conformation (left), or the RBD in the up conformation from the G614 S trimer in the one-RBD-up state (middle) or the RBD in the down conformation from the G614 S trimer in the one-RBD-up state (right) were colored according to local resolution estimated by cryoSPARC. (**C**) Representative density in gray surface from EM maps with a resolution better than 3.3Å, primarily focusing on the SP1-77 Fab and its binding interface with the RBD of the G614 S trimer.

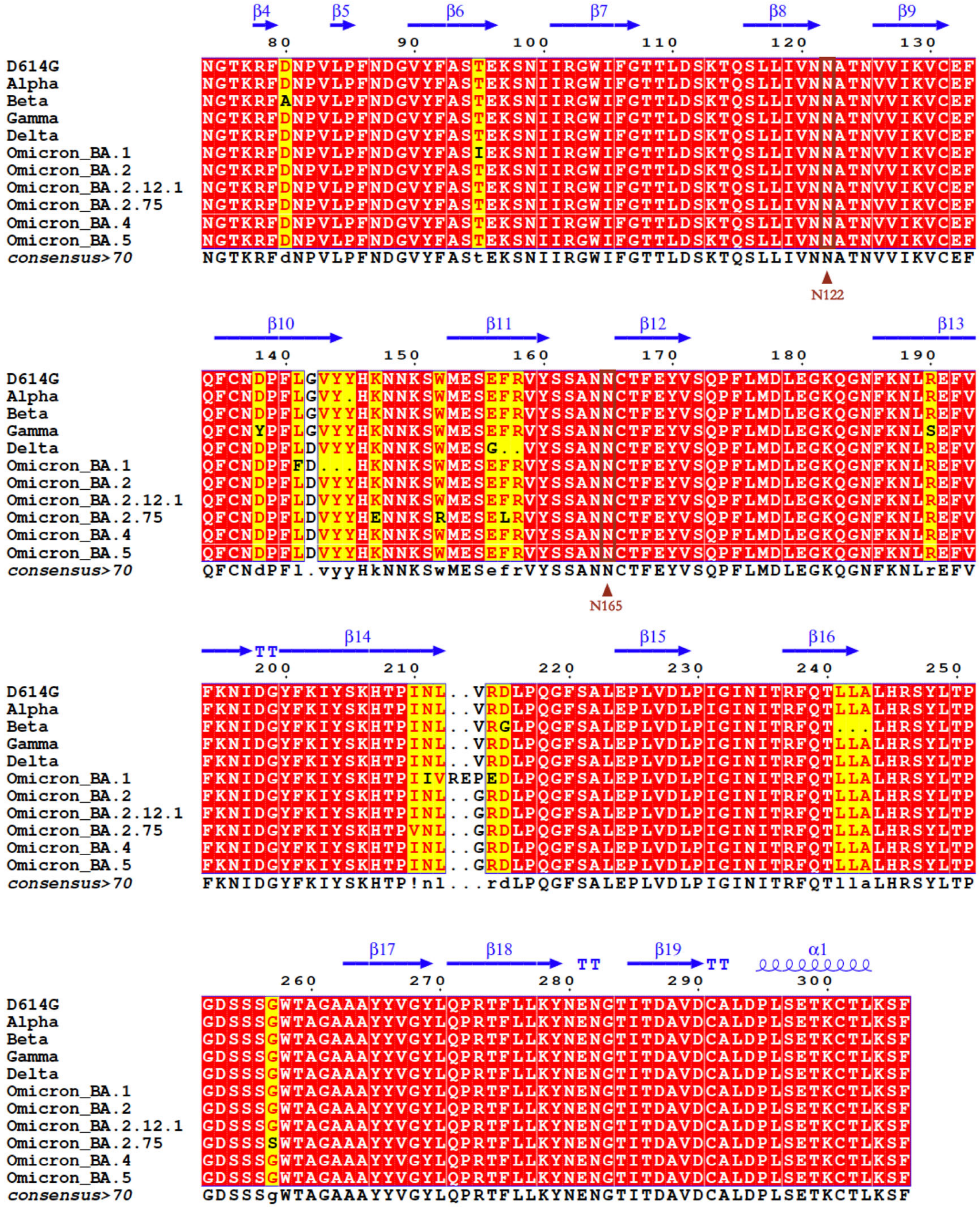

Figure S11. The NTD sequence alignment of SARS-CoV-2 variants. Amino acid sequence alignment of the NTD from SARS-CoV-2 variants. The sequences were aligned using ClustalW (*98*). Numbering was based on the sequence of the Wuhan-Hu-1 strain, as indicated on top of the alignment. Secondary structures for the NTD were also indicated in blue. The conserved residues were colored in red; the variations were highlighted in yellow. The two N-linked glycosylation sites (Asn122 and Asn165) in the NTD were marked in brown.

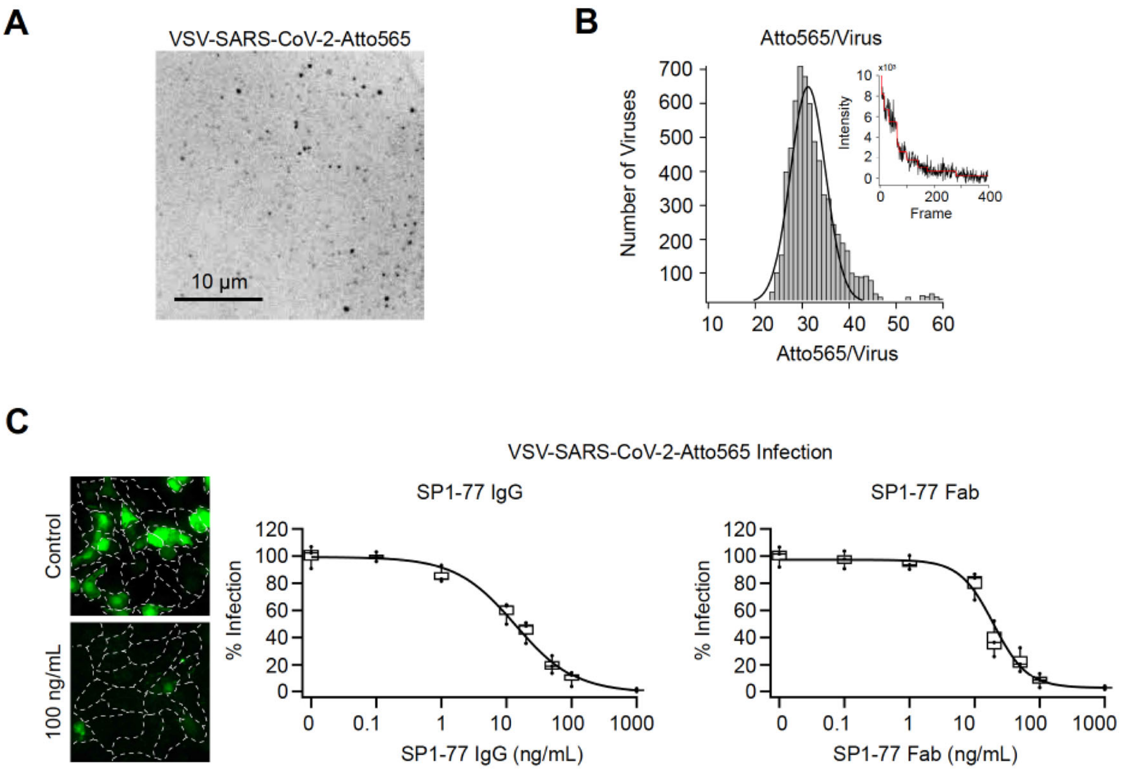

Figure S12. Single molecule calibration of VSV-SARS-CoV-2 Atto 565 virus. (**A**) Example image of Atto 565 fluorescence of virus adsorbed for 10 minutes on a glass coverslip coated with poly-D-lysine coated slides and imaged with spinning disc confocal microscopy. (**B**) Photobleaching used to determine the intensity of single Atto 565 dye intensities allowed to establish that ~20-40 dyes were attached to each virus. (**C**) Infection assays showing neutralization of VSV-SARS-CoV-2 Atto 565 infection with SP1-77 IgG or SP1-77 Fab. A soluble eGFP reporter genetically encoded into this VSV chimera allowed for infection to be determined by fluorescence imaging on a spinning disc confocal microscope as shown in the representative examples obtained 7 hour post infection. Cell outlines were obtained with a WGA-Alexa 647 membrane stain applied to cells immediately prior to fixation. Each condition was measured with 3 independent experiments. Data are shown in **Table S8**.
